# Physical Processing Controls the Structure, Mesoporosity, and Suspension-Phase Functionality of Nanostructured MnO_x_ Materials

**DOI:** 10.3390/nano16181141

**Published:** 2026-09-11

**Authors:** Ekaterina Saenko, Pavel Khramtsov, Igor Valtsifer, Anastasia Novokshonova, Viktor Valtsifer

**Affiliations:** 1 Institute of Technical Chemistry, Branch of the Perm Federal Research Center, Ural Branch of the Russian Academy of Sciences, 3 Akademika Koroleva St., Perm 614068, Russia; valtsifer.i@itcras.ru (I.V.); valtsifer.v@itcras.ru (V.V.); 2 Institute of Ecology and Genetics of Microorganisms, Branch of the Perm Federal Research Center, Ural Branch of the Russian Academy of Sciences, 13 Goleva St., Perm 614081, Russia; khramtsovpavel@yandex.ru (P.K.); anast218bio@gmail.com (A.N.)

**Keywords:** nanostructured manganese oxides, MnO_x_, mesoporous nanomaterials, physical processing, structure–property relationships, colloidal dispersion, nanozyme

## Abstract

Physical processing can modify the structural, textural, and dispersion characteristics of nanostructured oxide materials, thereby altering their functional state under suspension conditions. Here, poorly crystalline, sol–gel-derived porous MnO_x_ materials were used to establish how post-synthetic physical processing affects the relationship between nanoscale structure, accessible mesoporosity, powder-to-suspension transfer, and chromogenic response in 3,3′,5,5′-tetramethylbenzidine (TMB) oxidation. Two compositionally distinct processing series were examined: an ultrasonic processing/recovery route for Sr- and Fe-containing MnO_x_ and vibratory milling followed by identical ultrasonic dispersion for Sr-free Fe-containing MnO_x_. The recovered SrFeMn-US-S solid showed higher N_2_-accessible surface area and pore volume, stronger hydration signatures, and a larger low-temperature H_2_ temperature-programmed reduction (H_2_-TPR) contribution than SrFeMn-S. In contrast, vibratory milling of FeMn-S preserved the bulk Fe/Mn ratio but decreased *S_BET_* from 305.9 to 127.1 m^2^ g^−1^, *V_tot_* from 0.533 to 0.215 cm^3^ g^−1^, total H_2_ uptake from 0.38 to 0.34 mmol g^−1^, and the Mn concentration in the operationally defined stable suspension fraction from 50.5 to 17.3 mg L^−1^. At an identical assay concentration of 500 ng Mn mL^−1^, milled FeMn-S5 also exhibited a lower time-summed ΣA_652_ response than FeMn-S. Thus, milling affected both the efficiency of powder-to-suspension transfer and the Mn-normalized functional response of the dispersed material. The contrasting outcomes show that the functional state of nanostructured, powder-derived MnO_x_ is route-dependent and cannot be predicted from a single solid-state descriptor.

## 1. Introduction

Nanostructured manganese oxides (MnO_x_) are redox-active inorganic materials whose functional properties are strongly influenced by structural disorder, accessible surface area and porosity, and the state of the material under working conditions. They are capable of exhibiting oxidoreductase-like properties in the oxidation of organic substrates. One of the most widely used chromogenic model systems for evaluating this response is the oxidation of 3,3′,5,5′-tetramethylbenzidine (TMB), which produces a blue oxidized TMB product with a characteristic absorption band in the 652 nm region [1,2,3]. MnO_2_ and related MnO_x_ nanoparticles can oxidize chromogenic substrates under both H_2_O_2_-containing and H_2_O_2_-free conditions. Earlier studies on BSA-stabilized MnO_2_ nanoparticles implicated dissolved oxygen as an electron acceptor in oxidase-like reactions, whereas more recent work showed that TMB oxidation by manganese oxides may also proceed through an organic acid/aldehyde-mediated, O_2_-independent pathway under weakly acidic or neutral conditions [4,5]. This mechanistic diversity is important because the measured chromogenic response depends not only on oxide composition but also on the reaction medium and the state of the dispersed material. TMB remains a classical chromogenic substrate in peroxidase-based chemical and biochemical analysis [6]. These reactions have made MnO_x_ a widely used model platform for evaluating structure- and environment-dependent oxidoreductase-like behavior in dispersed oxide systems [7,8,9]. MnO_2_- and MnO_x_-based nanozymes have also been applied as oxidase- and peroxidase-mimicking systems in both H_2_O_2_-containing and H_2_O_2_-free assays [10,11,12,13,14]. Related transition-metal oxide nanozymes are widely used in POX- and OX-like chromogenic tests, including mesoporous Fe_2_O_3_, Co-Fe oxides, CuO, perovskites, spinels, and mixed-oxide Co_3_O_4_/MO_3_ systems [15,16,17,18,19,20].

Post-synthetic physical processing can alter nanostructured functional oxide materials beyond simple particle-size reduction. Mechanical treatment may modify accessible porosity, local structural order, redox-active states, and interparticle contacts, whereas ultrasonic treatment can alter aggregation, surface accessibility, and transfer of the solid into the liquid phase [21,22,23,24]. For porous and poorly crystalline oxide materials, such processing-induced changes are particularly relevant because the functional material present under suspension conditions may not retain the same accessible structural and textural characteristics as the initial dry powder. However, these effects are often discussed separately, and the relationship between solid-state changes, suspension formation, and functional response remains insufficiently resolved for powder-derived MnO_x_ systems.

Unlike soluble enzymes, nanozymes function as heterogeneous catalysts; therefore, their measured response depends not only on the chemical nature of the active centers but also on surface accessibility, aggregation state, reaction-medium composition, chemical transformations of the material, and colloidal stability of the dispersed system. For powdered oxide nanozymes, the fraction present under assay conditions may therefore differ from the material characterized in the dry state. Panferov et al. showed that multiple activities, colloidal instability, and chemical transformations complicate the kinetic interpretation of oxidoreductase-mimicking nanozymes [3]. In addition, Dong et al. demonstrated that the peroxidase-like activity of Fe_3_O_4_ can be accompanied by chemical transformation of the material, whereas for Prussian Blue nanozymes, colloidal stability and aggregation were shown to be linked to peroxidase-like activity [25,26].

Despite these considerations, powder-to-working-suspension preparation has rarely been treated as an independently controlled variable in nanozyme assays. Differences in apparent nanozyme response may therefore reflect not only intrinsic properties of the oxide surface but also dispersion efficiency, aggregate removal, sedimentation behavior, and the amount of material that remains accessible in the working suspension. A representative literature survey together with the corresponding references is provided in Table S1 (Supplementary Information). Across the selected studies, assay composition and catalyst loading are generally reported more consistently than dispersion parameters, aggregate-removal procedures, sedimentation control, DLS/PDI, ξ-potential, and stability in the reaction medium. More complete characterization and reporting of the powder-to-suspension transition is therefore required to distinguish intrinsic solid-state properties from processing-dependent changes in the dispersed functional material [26,27].

In the present work, this methodological problem is examined using powdered sol–gel-derived porous, poorly crystalline MnO_x_ materials as a model platform. The objective was not to maximize the apparent TMB response of MnO_x_ nanozymes, but to determine how physical post-synthetic treatment controls the structural and textural state of the material and the fraction of a powder-derived oxide that becomes accessible under suspension assay conditions. Specifically, we examined how ultrasonic treatment and vibratory milling affect N_2_-accessible mesoporosity, hydration, reducibility, transfer of MnO_x_ material into a stable dispersed fraction, and the Mn-normalized A_652_ response in the TMB oxidation reaction. The central hypothesis was that physical treatment is neither a neutral preparation step nor a universally activating operation, but modifies the state of MnO_x_ that becomes accessible under suspension conditions. Accordingly, the functional response was considered in terms of the sequence “initial powder ⟶ physical processing ⟶ suspension formation ⟶ suspension-accessible MnO_x_ state ⟶ A_652_ response”. This approach provides an experimental framework for linking processing-induced changes in solid-state descriptors to the formation of the dispersed functional material and for evaluating how the complete processing history governs the suspension-phase functional state of porous, nanostructured MnO_x_ materials.

## 2. Materials and Methods

### 2.1. Materials

Potassium permanganate (KMnO_4_, REAKHIM LLC, Moscow, Russia), maleic acid (REAKHIM LLC, Moscow, Russia), iron(III) chloride hexahydrate (FeCl_3_·6H_2_O, PanReac AppliChem, Castellar del Vallès, Barcelona, Spain), sodium hydroxide (NaOH, ECOS-1, Moscow, Russia), strontium chloride hexahydrate (SrCl_2_·6H_2_O, Unikhim, Saint Petersburg, Russia), nitric acid (HNO_3_, SigmaTek, Khimki, Russia), hydrochloric acid (HCl, BSK, Ufa, Russia), sodium citrate (Vekton, Moscow, Russia), propionic acid (Macklin, Shanghai, China), 3,3′,5,5′-tetramethylbenzidine (TMB, Macklin, Shanghai, China), dimethyl sulfoxide (DMSO, Usolye-Siberian Chemical and Pharmaceutical Plant, Usolye-Siberian, Russia), and deionized water were used without additional purification. TMB stock solutions were prepared in DMSO. Low-ionic-strength sodium propionate medium was prepared by adjusting propionic acid solution to the required pH with NaOH and/or HCl.

### 2.2. Synthesis of Powders

MnO_x_ materials were synthesized by a sol–gel route based on the redox reaction between KMnO_4_ and maleic acid. Aqueous solutions of 0.05 M maleic acid and 0.15 M KMnO_4_ were mixed at room temperature under vigorous stirring until formation of a dark brown gel. For Fe-containing samples, FeCl_3_·6H_2_O was added to the initial reaction mixture before gelation in an amount corresponding to 5 mol% Fe relative to the calculated Mn content. The obtained gel was washed with deionized water to remove soluble by-products and dried in air at 105 °C. For preparation of Sr-containing samples, the oxide precursor was first converted into the Na-form by treatment with 0.2 M NaOH solution until ion-exchange equilibrium was reached, as indicated by pH stabilization. The material was then washed with deionized water to approximately neutral pH and treated with 0.2 M SrCl_2_ solution to introduce Sr^2+^. The samples were dried in air and thermally treated at 150 °C for 6 h. The sample designations used throughout the manuscript are as follows: Mn-S, Fe- and Sr-free MnO_x_; SrMn-S, Sr-containing Fe-free MnO_x_; FeMn-S, Fe-containing Sr-free MnO_x_; SrFeMn-S, Sr- and Fe-containing MnO_x_; FeMn-S5, FeMn-S after five vibratory-milling stages; and SrFeMn-US-S, the SrFeMn-S-derived material associated with the citrate-assisted ultrasonic processing route. Depending on the experiment, SrFeMn-US-S was characterized either in the suspension state or as the recovered dry solid obtained through the concentrated processing/recovery route. Independently prepared batches of parent materials with the same nominal SrMn-S and SrFeMn-S compositions were previously examined in the context of cation-directed post-synthetic transformations [28].

### 2.3. Vibratory Ball Milling

The FeMn-S5 sample was prepared by dry vibratory ball milling of the parent FeMn-S powder using a GT600 Multi high-energy ball mill (Beijing Grinder Instrument Co., Ltd., Beijing, China). In a typical procedure, 1.0 g of FeMn-S powder was loaded into a 50 mL stainless-steel jar containing one 25 mm stainless-steel ball. Five 10 min milling stages were applied at 1000 rpm with 30 min cooling intervals between stages. FeMn-S5 was obtained after five consecutive milling stages, corresponding to a total milling time of 50 min. The treatment was performed under dry conditions without the addition of a solvent.

### 2.4. Material Characterization

Elemental analysis of liquid and solid samples was carried out by atomic absorption spectrometry on an iCE 3500 instrument (Thermo Fisher Scientific, Waltham, MA, USA). For solid samples, 10 mg portions were digested in a mixture of concentrated HCl and HNO_3_ (1:1, *v*/*v*), evaporated, dissolved in deionized water, and quantitatively transferred into 50 mL volumetric flasks. For suspension samples, 10 mL aliquots were treated in the same manner. The elemental contents were calculated from the AAS-measured concentrations taking into account the final solution volume and the initial sample mass or suspension aliquot volume; values for solid samples were expressed as wt.%, whereas suspension concentrations were expressed as mg L^−1^.

Powder X-ray diffraction (XRD) patterns were recorded on an XRD-7000 diffractometer (Shimadzu Corporation, Kyoto, Japan) using Cu Kα radiation over the 2θ range of 5–80°. Qualitative reference-pattern matching was performed using the Search/Match procedure with the PDF-2 database. Because of the broad and overlapping diffraction features, the resulting PDF-2 matches were treated as candidate structural matches rather than as unambiguous phase assignments. For the determination of the full width at half maximum (FWHM), two representative broad diffraction features centered at approximately 37° and 66° 2θ were fitted independently using pseudo-Voigt profiles with local linear backgrounds in Microsoft Excel Solver using the GRG Nonlinear algorithm. Fixed fitting windows of 33.5–40.0° and 61.0–71.0° 2θ, respectively, were used for all samples. The original unsmoothed XRD data were used for fitting. The FWHM values obtained from the two diffraction features were analyzed separately and were not averaged.

Apparent coherence lengths were estimated from the Scherrer equation [29]:

$$D_{app}=\frac{K\lambda }{\beta cos\theta },$$

where *D_app_* is the apparent coherence length, *K* = 0.9 is the shape factor, *λ* = 1.5406 Å is the wavelength of Cu Kα radiation, *β* is the full width at half maximum of the diffraction peak expressed in radians, and *θ* is the Bragg angle. Because the diffraction maxima were broad and overlapping and instrumental broadening was not independently corrected, the resulting *D_app_* values are reported only as apparent broadening-derived coherence-length estimates and are not interpreted as physical particle diameters.

Raman spectra were collected at room temperature on a Senterra Raman spectrometer (Bruker Optics GmbH, Ettlingen, Germany) in the range of 100–4000 cm^−1^ using a 532 nm excitation laser.

The FTIR spectra were recorded using a VERTEX-80v Fourier-IR spectrometer (Bruker Optics GmbH, Ettlingen, Germany) in the range of wave numbers 4000–400 cm^−1^ with a spectral resolution of 2 cm^−1^. The relative contribution of hydrated and hydroxylated states was evaluated from the FTIR spectra using relative spectral descriptors the *I_H_*_2*O*_/*I_Mn-O_* and *I_OH_*/*I_Mn-O_* ratios. The intensities *I_OH_*, *I_H_*_2*O*_, and *I_Mn-O_* were determined as the maximum band intensities in the 3000–3700, 1500–1700, and 450–650 cm^−1^ regions, respectively. These ratios were used only as semi-quantitative within-series spectral descriptors and were not interpreted as absolute measures of surface hydroxyl or water coverage.

Thermal behavior was studied by simultaneous thermogravimetric analysis and differential scanning calorimetry (TGA/DSC) using a TGA/DSC 1/1100 LF instrument (Mettler-Toledo GmbH, Greifensee, Switzerland). Measurements were performed at a heating rate of 10 °C min^−1^; prior to analysis, the samples were dried at 105 °C to constant weight.

Textural properties were determined from nitrogen adsorption–desorption isotherms measured at −196 °C on an ASAP 2020 instrument (Micromeritics Instrument Corporation, Norcross, GA, USA) after degassing under vacuum at 105 °C for 3 h. The specific surface area was calculated by the Brunauer–Emmett–Teller (BET) method, and the pore size distribution was derived from the desorption branch of the isotherms using the Barrett–Joyner–Halenda (BJH) model [30,31]. The BJH-derived pore-size distributions were used as comparative descriptors across the sample series and were not interpreted as absolute geometric pore dimensions.

Reduction behavior and total H_2_ consumption were evaluated by H_2_ temperature-programmed reduction (H_2_-TPR) using a Chemosorb analyzer (Neosib, Novosibirsk, Russia). Prior to the measurements, samples (ca. 0.1 g) were pretreated in a nitrogen flow at 100 °C for 1 h to remove adsorbed surface species. H_2_-TPR was performed over the temperature range of 20–750 °C at a linear heating rate of 5 °C min^−1^. Total H_2_ uptake was determined from the integrated TCD response using the instrument calibration for the 5 vol % H_2_/Ar mixture and was normalized to the sample mass.

### 2.5. Preparation of Operationally Defined Stable MnO_x_ Suspension Fractions

Operationally defined stable MnO_x_ suspension fractions were prepared by probe ultrasonication followed by removal of large aggregates. The powder was first gently ground in a porcelain mortar. Then, 40 mg of the fine powder was added to 40 mL of 0.1 mol L^−1^ sodium citrate in a 50 mL plastic centrifuge tube and sonicated on ice for 4 h using a horn probe. The suspension was centrifuged at 10,000× *g* for 10 min, and the supernatant was removed. Deionized water, 40 mL, was added to the sediment; the suspension was sonicated for 2 min and centrifuged again at 10,000× *g* for 10 min. The supernatant was then collected and centrifuged at 100× *g* for 30 min to remove large aggregates. The final supernatant was used as the stable suspension fraction and stored at +4 °C until analysis. This protocol corresponds to the route used for forming the working MnO_x_ suspensions in which A_652_ was measured.

For preparation of the concentrated ultrasonic fraction SrFeMn-US-S, a tenfold larger amount of SrFeMn-S powder was processed to obtain sufficient material for structural and textural characterization.

In this case, 400 mg of the material was ultrasonically treated in sodium citrate medium as ten 40 mg portions. The resulting suspension was centrifuged at 10,000× *g* for 10 min. The sediment was resuspended in 1 mL of deionized water, vortexed, and centrifuged at 100× *g* for 1 min to collect suspension droplets. The combined concentrated suspension was further sonicated on ice for 20 min using a 3 mm horn probe at 60% amplitude, dialyzed twice against deionized water, and then dried at room temperature to obtain the solid SrFeMn-US-S product for powder characterization.

Probe sonication was performed using a SONICS MATERIAL VCX 130 (Sonics & Materials, Inc., Newtown, CT, USA) ultrasonic processor operating at 20 kHz and a nominal maximum power of 130 W. A 6-mm titanium probe was used at 60% amplitude in continuous mode. Samples were maintained in an ice bath, and the suspension temperature was kept below 14 °C.

Hydrodynamic diameters (D_h_) of the dispersed MnO_x_ fractions were measured in both water and the catalytic medium to assess colloidal stability. Aliquots (0.7 mL) of the suspensions were briefly sonicated (3 mm probe, 60% amplitude, 30 s) and then diluted to a final concentration of 1 µg Mn·mL^−1^ in a 9:1 mixture of 1 mmol·L^−1^ sodium propionate buffer (pH 3.8–5.8) and DMSO. Samples were incubated at room temperature for 10 min prior to measurement. A viscosity value corresponding to 10% (*v*/*v*) DMSO in water was used for data processing. Three technical replicates were acquired for each sample.

Zeta-potential measurements were performed using a BeNano 180 Zeta Pro instrument (Bettersize Instruments, Dandong, Liaoning, China). Aliquots (0.7 mL) of the suspensions were briefly sonicated (3 mm probe, 60% amplitude, 30 s) and then diluted to a final concentration of 1 μg Mn mL^−1^ in 1 mmol L^−1^ sodium propionate buffer over the pH range 3.8–5.8. Three technical replicates were acquired for each sample at each pH value.

### 2.6. TMB Oxidation Assay and Chromogenic Response

Effect of pH: To each well of a flat-bottom 96-well plate, 160 µL of 1 mmol L^−1^ sodium propionate buffer (pH 3.8–5.8) and 20 µL of TMB solution in DMSO (4 mmol L^−1^) were added. Both the buffer and TMB solution were pre-warmed to 37 °C. The plate was equilibrated in the temperature-controlled block of the spectrophotometer for 5 min, after which 20 µL of an aqueous MnO_x_ suspension fraction was added and the contents were rapidly mixed. The final Mn concentration in the reaction mixture was 500 ng Mn mL^−1^, corresponding to 9.1 µmol Mn L^−1^. Thus, all catalytic comparisons were performed under Mn-normalized conditions. Absorbance at 652 nm was recorded every 10 s for 10 min. Each condition was measured in triplicate, and the absorbance of a TMB-containing blank without nanozyme was subtracted from the corresponding measurements.

The time-summed chromogenic index, ΣA_652_, was calculated as the discrete sum of the blank-corrected A_652_ values acquired every 10 s over the 10 min observation period. Because the numerical value of ΣA_652_ depends on the acquisition interval and observation time, this operational index was used only for within-study comparisons performed under identical measurement conditions.

Effect of TMB concentration: The effect of substrate concentration was examined at pH 3.8 using TMB stock solutions in DMSO with concentrations ranging from 0.25 to 32 mmol L^−1^. The reaction mixtures were prepared using the same volumes and procedure described above, giving a final concentration of 500 ng Mn mL^−1^. Absorbance measurements and blank correction were performed as described above. For each TMB concentration, ΣA_652_ was calculated using the procedure defined above.

## 3. Results and Discussion

The study design was aimed at distinguishing three operational states of the sol–gel MnO_x_ materials: the initial powder, the solid fraction or powder-like state obtained after physical treatment, and the working suspension in which the A_652_ response is formed. This approach accounts for the fact that powder characterization methods describe the material in the dry state, whereas the catalytic response is determined by the state of MnO_x_ after physical treatment and dispersion in the liquid reaction medium.

In the first series, the initial Sr- and Fe-containing powder, SrFeMn-S, was converted into a stable suspension by ultrasonic dispersion, and its Mn-normalized A_652_ response was evaluated. In parallel, sufficient solid material for powder-state characterization was recovered through a concentrated citrate-assisted ultrasonic processing route involving additional sonication, dialysis, and drying. The resulting SrFeMn-US-S solid was therefore treated as a processing-route-associated analogue rather than as a dried replica of the working catalytic suspension. Comparison of SrFeMn-S and SrFeMn-US-S was used to identify structural, textural, thermal, redox changes associated with ultrasonic processing and recovery.

In the second series, a different experimental route was used: the Fe-containing Sr-free sample FeMn-S was subjected to preliminary vibratory milling to obtain FeMn-S5, after which both powders were converted into suspensions by ultrasonic dispersion. The A_652_ response was compared for suspensions prepared from the initial and milled powders. The FeMn-S/FeMn-S5 pair enabled evaluation of whether preliminary mechanical milling promotes the formation of a functional ultrasonically dispersed suspension or, conversely, limits the transfer of the material into the operationally defined stable dispersed fraction.

**Figure 1:**
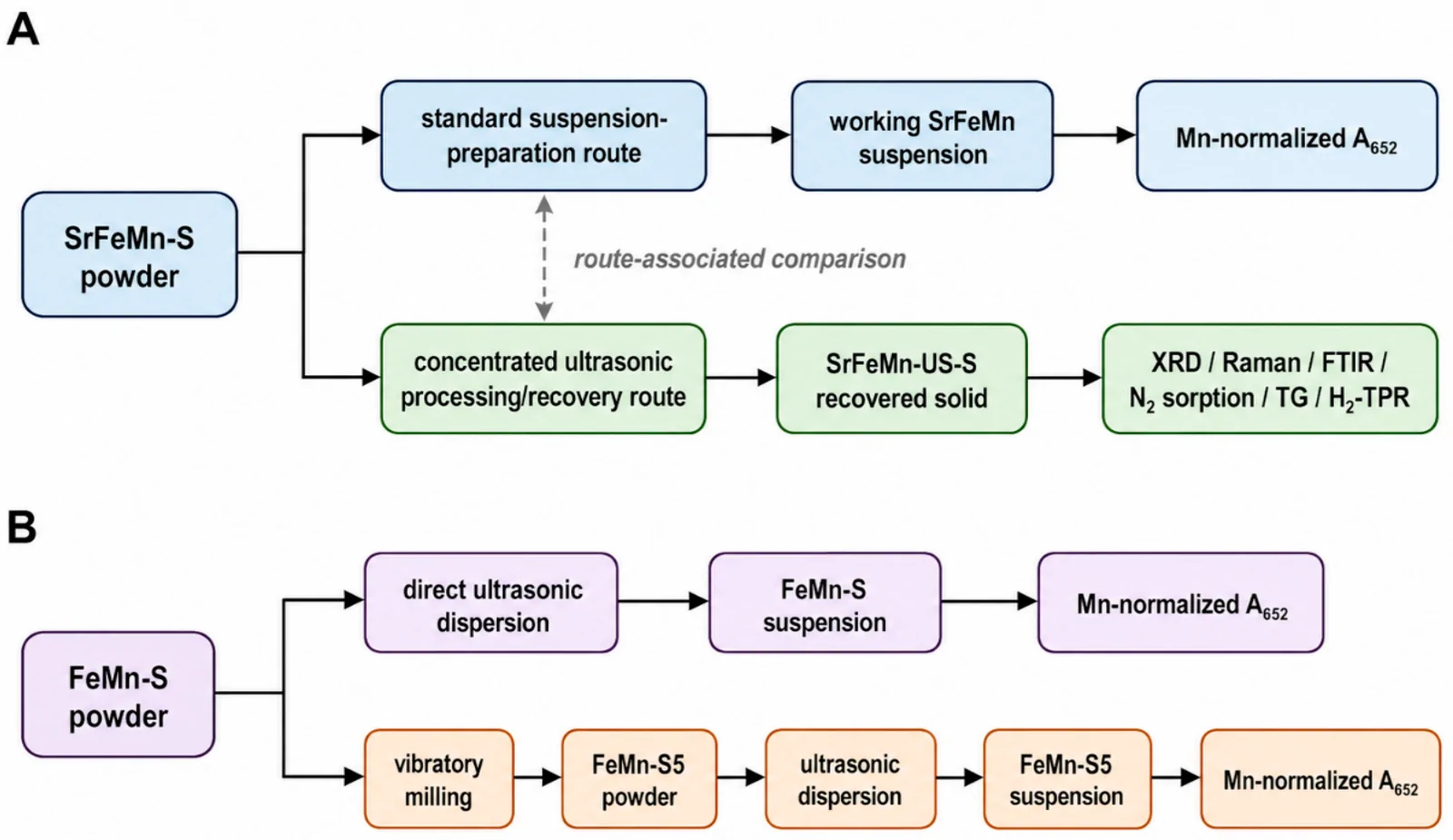
Schematic representation of the experimental routes used for sample processing and analysis. (**A**) Two related ultrasonic-processing routes applied to SrFeMn-S: preparation of the working suspension for Mn-normalized A_652_ measurements and a concentrated ultrasonic processing/recovery route used to obtain the SrFeMn-US-S solid for powder-state characterization. (**B**) Comparison of two routes for preparing FeMn-S suspensions: direct ultrasonic dispersion and vibratory milling followed by ultrasonic dispersion, with subsequent evaluation of the Mn-normalized A_652_ response.

The Fe-free Mn-S and SrMn-S samples were included as reference materials to provide compositional and processing context for the effects associated with Fe incorporation, Sr incorporation, and mild thermal treatment on the dry-state properties of the MnO_x_ matrix. They were not used as full catalytic-comparison samples in the Mn-normalized A_652_ analysis, which was focused on the Fe-containing processing routes that produced the stable suspension fractions discussed below. The overall study design is shown in Figure 1. These processing series were considered as complementary rather than directly equivalent scenarios. Their purpose was to determine whether physical processing produces a uniform activating effect or instead leads to material- and route-dependent outcomes.

The Fe/Mn and Sr/Mn molar ratios calculated from elemental analysis are summarized in Table 1. For the SrFeMn-S/SrFeMn-US-S pair, the Fe/Mn ratio remains relatively close after ultrasonic treatment, decreasing only from 0.0694 for SrFeMn-S to 0.0615 for SrFeMn-US-S. In contrast, the Sr/Mn ratio decreases markedly from 0.3339 to 0.1304. Thus, SrFeMn-US-S is characterized by a lower Sr/Mn ratio than the initial SrFeMn-S, while retaining the Fe-containing character of the MnO_x_ matrix.

Vibratory milling has only a minor effect on the bulk Fe/Mn composition of the powder. The Mn contents are 28.62 wt% for FeMn-S and 28.90 wt% for FeMn-S5, while the Fe contents are 5.03 and 5.22 wt%, respectively. The corresponding Fe/Mn molar ratios are also close: 0.1728 for FeMn-S and 0.1777 for FeMn-S5. Therefore, the subsequent differences between these two samples cannot be attributed to a change in the bulk Fe/Mn composition of the solid phase.

**Figure 2:**
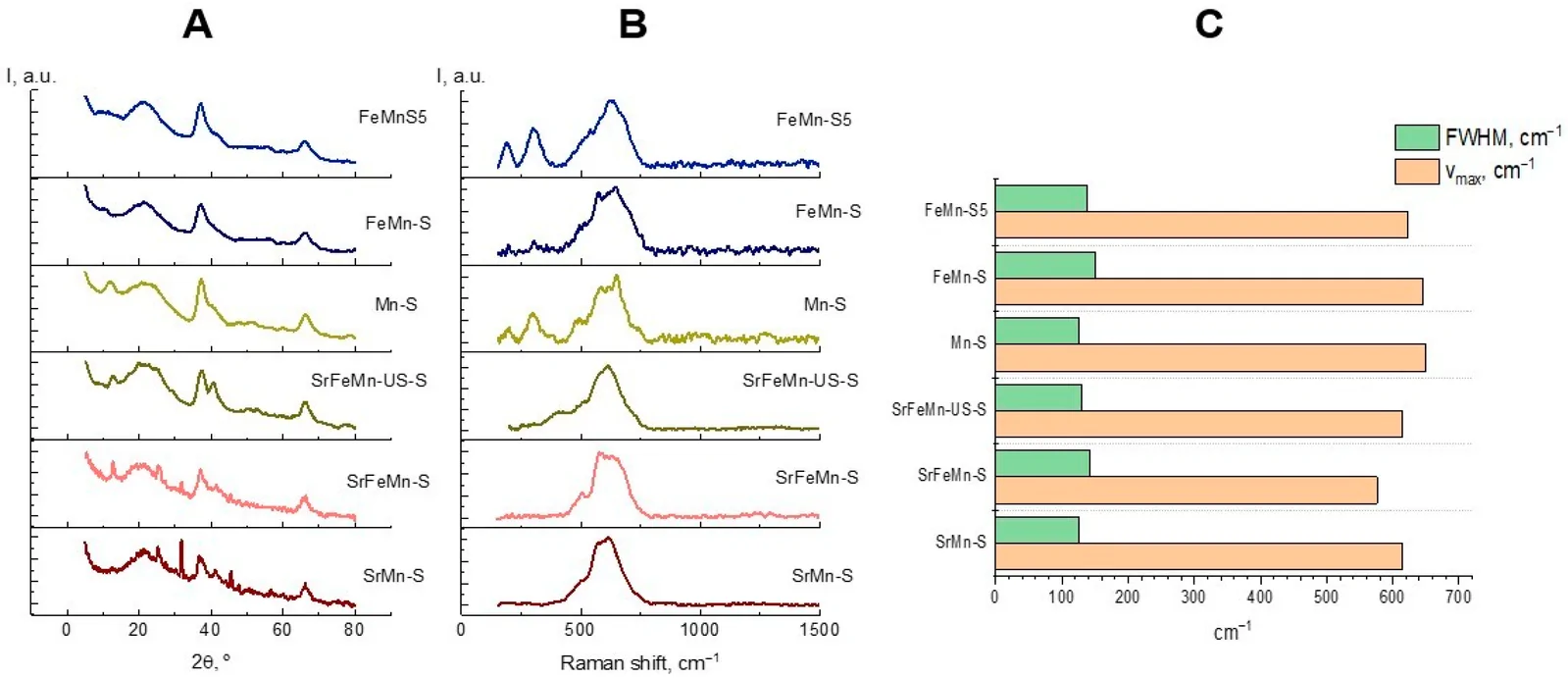
Structural and vibrational characterization of the MnO_x_ samples. (**A**) XRD patterns and (**B**) Raman spectra of SrMn-S, SrFeMn-S, SrFeMn-US-S, Mn-S, FeMn-S, and FeMn-S5. (**C**) Position of the maximum of the main Mn-O Raman envelope (ν_max_) and its full width at half maximum (FWHM). The curves are vertically shifted for clarity.

The X-ray diffraction patterns of all samples are characterized by broad, low-intensity maxima and a pronounced diffuse background, consistent with a poorly ordered or nanocrystalline state of the MnO_x_ matrix rather than the formation of well-crystallized individual phases (Figure 2A). The broad diffraction features near 37° and 66° 2θ were analyzed independently, and the corresponding Scherrer broadening-derived apparent coherence lengths ranged from approximately 2.7 to 4.6 nm, depending on the sample and diffraction feature (Table S2). These values should be regarded as estimates, because instrumental broadening was not subtracted and the maxima themselves correspond to a poorly ordered MnO_x_ matrix.

Qualitative Search/Match analysis produced several overlapping matches with manganese oxide and oxyhydroxide reference patterns. The corresponding candidate PDF-2 card numbers are provided in Table S2. However, owing to the broad diffraction maxima and pronounced diffuse scattering, unambiguous phase assignment is not possible. For the same reason, assignment of the individual broad features to specific (hkl) reflections was not considered sufficiently reliable. The XRD data are therefore interpreted conservatively as evidence of a poorly ordered MnO_x_/Mn-O-H matrix with short-range structural heterogeneity rather than as a resolved mixture of discrete crystalline phases. Changes in the relative intensities and positions of the broad maxima indicate a redistribution of short-range order and are consistent with variations in local Mn-O/Mn-O-H environments within a defective, poorly crystalline matrix [32,33,34]. XRD alone does not establish a specific hydration-induced phase transformation. Sr introduction, Fe addition, and mild thermal treatment at 150 °C modify the width, relative intensity, and position of the broad diffraction features but do not produce a consistent set of new sharp reflections that would permit unambiguous identification of a separate well-crystallized phase.

For the SrFeMn-S/SrFeMn-US-S pair, the analyzed broad-feature centers shift from approximately 37.08° and 66.03° for SrFeMn-S to 37.28° and 66.22° 2θ for SrFeMn-US-S, respectively. Qualitative Search/Match analysis yields overlapping coincidences with Mn oxide, oxyhydroxide, and hydrated Mn-O reference patterns. For SrFeMn-US-S, additional or more prominent coincidences with hydrated Mn-O/Mn-O-H reference patterns are observed. Given the breadth and overlap of the diffraction maxima, these coincidences are not regarded as definitive phase assignments. The changes are therefore interpreted as redistribution of short-range Mn-O/Mn-O-H environments within a defective, poorly ordered matrix rather than as evidence of a resolved phase transformation.

For the FeMn-S/FeMn-S5 pair, the overall XRD profile after vibratory milling remains close to that of the initial sample. The positions of the two analyzed broad features remain essentially unchanged after milling (37.07° versus 37.02° and 66.18° versus 66.16° 2θ for FeMn-S and FeMn-S5, respectively). Qualitative Search/Match analysis yields broadly similar overlapping coincidences with Mn oxide and Mn oxyhydroxide/hydroxide reference patterns for FeMn-S and FeMn-S5. Thus, vibratory milling does not induce a phase transformation that can be reliably detected by XRD. The observed differences are limited mainly to subtle variations in the width and relative intensity of the broad diffraction features, consistent with changes in short-range structural ordering rather than with formation of a new crystalline phase.

The Raman spectra of all samples contain broad, overlapping bands in the 500–650 cm^−1^ region, which is characteristic of Mn-O vibrations in MnO_6_ octahedral environments (Figure 2B) [35,36]. These broad envelopes are consistent with a low degree of long-range order and a distribution of local Mn-O states, in agreement with the XRD data. In the Sr-containing series, SrFeMn-S shows the maximum of the main Mn-O envelope at approximately 576 cm^−1^ with an FWHM of about 142 cm^−1^. After ultrasonic treatment, the maximum for SrFeMn-US-S shifts to 613.5 cm^−1^, while the FWHM decreases to 128.9 cm^−1^ (Figure 2C). The shift in ν_max_, together with the decrease in FWHM, is consistent with a redistribution of overlapping Mn-O vibrational contributions and changes in local short-range ordering. Because the Raman feature is broad and strongly overlapping, the shift is not assigned to a single vibrational mode or a specific Mn oxidation state. The independent FTIR and TG results discussed below indicate that the recovered SrFeMn-US-S solid also contains a larger relative contribution of water/OH-related states [34].

In the Fe-containing Sr-free series, FeMn-S exhibits the broadest main Mn-O Raman envelope among the studied samples, consistent with a broad distribution of overlapping Mn-O vibrational contributions and local structural disorder. After milling to obtain FeMn-S5, the maximum shifts to 622.0 cm^−1^, and the FWHM decreases to 137.6 cm^−1^. At the same time, no new sharp Raman bands appear that could be assigned to a separate crystalline Fe oxide or another individual phase. Together, the XRD and Raman data indicate preservation of the poorly crystalline MnO_x_ framework accompanied by route-dependent reorganization of local Mn-O/Mn-O-H environments. Defect-related features such as oxygen vacancies, local lattice distortions, and disordered surface regions are known to modify the electronic and redox properties of transition-metal oxides and can consequently influence their catalytic response [37,38,39]. However, the broad XRD and Raman features observed here provide evidence of structural disorder only and do not allow specific defect types to be identified or quantified unambiguously. Therefore, oxygen vacancies and related defect states are considered possible contributors to the processing-dependent behavior of the present MnO_x_ materials rather than experimentally established descriptors.

**Figure 3:**
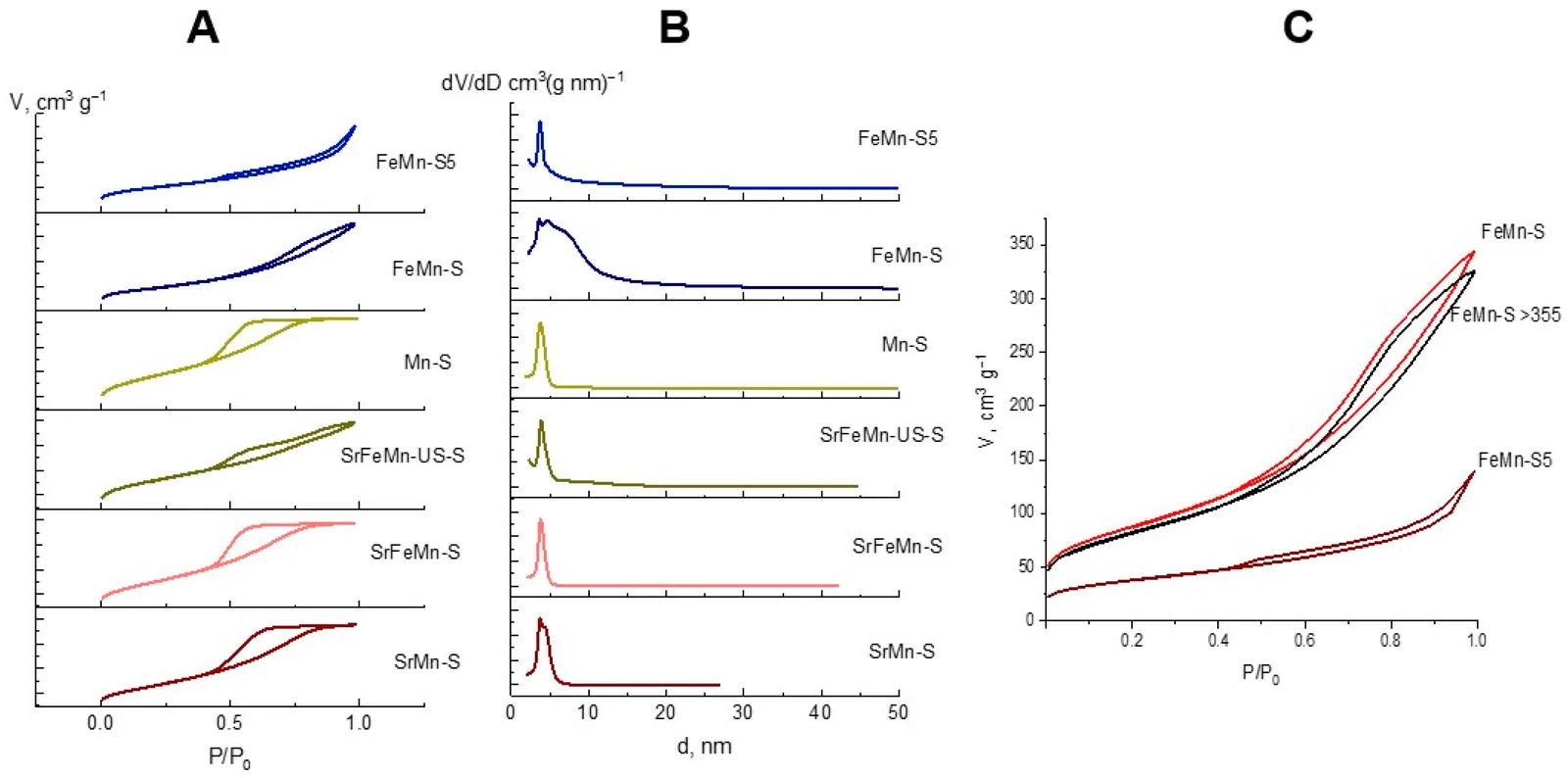
Textural characterization of the samples based on low-temperature nitrogen adsorption data. (**A**) N_2_ adsorption–desorption isotherms of SrMn-S, SrFeMn-S, SrFeMn-US-S, Mn-S, FeMn-S, and FeMn-S5. (**B**) BJH pore-size distributions derived from the desorption branches of the isotherms. (**C**) Comparison of the isotherms of FeMn-S, the FeMn-S > 355 µm fraction, and mechanically milled FeMn-S5. The curves are vertically shifted for clarity.

Low-temperature nitrogen adsorption and H_2_-TPR were used to compare the accessible mesoporosity and reduction behavior of the studied materials. The BJH-derived pore-size distributions are predominantly located in the mesopore range, with characteristic pore widths of approximately 4–7 nm, i.e., within the mesopore region according to the IUPAC classification [30]. The specific surface area, total pore volume, and H_2_ uptake vary with composition and the type of physical treatment (Figure 3 and Table 2).

In the Sr-containing series, SrMn-S has *S_BET_* = 131.0 m^2^ g^−1^ and *V_tot_* = 0.165 cm^3^ g^−1^. SrFeMn-S shows a moderately higher *S_BET_* than SrMn-S, while their *V_tot_* values remain similar (Table 2).

The most pronounced textural difference in this series is observed for the SrFeMn-US-S solid recovered after the concentrated ultrasonic processing route. For SrFeMn-US-S, *S_BET_* increases to 241.5 m^2^ g^−1^, and *V_tot_* increases to 0.277 cm^3^ g^−1^. Relative to SrFeMn-S, this corresponds to increases of 60.2% in specific surface area and 64.8% in total pore volume. At the same time, the average pore width remains nearly unchanged: 4.5 nm for SrFeMn-S and 4.6 nm for SrFeMn-US-S. Therefore, relative to the parent SrFeMn-S powder, the recovered SrFeMn-US-S solid exhibits substantially higher N_2_-accessible surface area and pore volume. Because this processing route also involved citrate exposure, fraction recovery, Sr depletion, dialysis, and drying, the individual contributions of these steps cannot be separated within the present experimental design. Therefore, the observed textural differences are attributed to the overall ultrasonic processing/recovery route rather than to ultrasound alone.

Within the Sr-free series, FeMn-S exhibits substantially higher *S_BET_* and *V_tot_* than Mn-S. Mn-S has *S_BET_* = 237.0 m^2^ g^−1^ and *V_tot_* = 0.238 cm^3^ g^−1^, whereas FeMn-S exhibits the highest values among the studied samples: *S_BET_* = 305.9 m^2^ g^−1^ and *V_tot_* = 0.533 cm^3^ g^−1^. Within the Sr-free series, FeMn-S exhibits a 29.0% higher *S_BET_* and a 123.5% higher *V_tot_* than Mn-S. These route-associated differences indicate a more developed N_2_-accessible mesoporous texture in FeMn-S.

The >355 µm fraction retained textural and H_2_-TPR characteristics close to the unfractionated FeMn-S powder. Thus, simple coarse-fraction separation within the parent FeMn-S material produced substantially smaller changes than vibratory milling. This control does not isolate particle size as an independent variable but indicates that the milling-induced changes are not reproduced by coarse fractionation alone. Its *S_BET_* and *V_tot_* were only 4.8 and 5.3% lower, respectively, while the average pore width remained nearly unchanged (Table 2).

In contrast, vibratory milling causes a pronounced loss of accessible mesoporosity. *S_BET_* decreases from 305.9 to 127.1 m^2^ g^−1^, corresponding to a 58.5% decrease, while *V_tot_* decreases from 0.533 to 0.215 cm^3^ g^−1^, corresponding to a 59.6% decrease. At the same time, the average pore width remains close to that of FeMn-S: 6.8 nm for FeMn-S5 versus 7.0 nm for FeMn-S. This means that milling does not simply reduce the average mesopore diameter but mainly decreases the overall accessible surface area and pore volume. This behavior may reflect partial loss of accessible interparticle porosity, aggregate densification, and/or blockage of mesoporous pathways during mechanical treatment [40,41]. However, these mechanisms cannot be directly resolved from the present data and are therefore considered possible interpretations of the nitrogen-adsorption results.

The two processing routes produced contrasting changes in N_2_-accessible mesoporosity within their respective series. The concentrated ultrasonic processing/recovery route increased *S_BET_* and *V_tot_* for the recovered Sr-containing solid, whereas vibratory milling markedly decreased both parameters for FeMn-S. Physical processing therefore did not impose a common textural trajectory on the studied MnO_x_ materials and cannot be regarded a priori as a universally activating operation.

**Figure 4:**
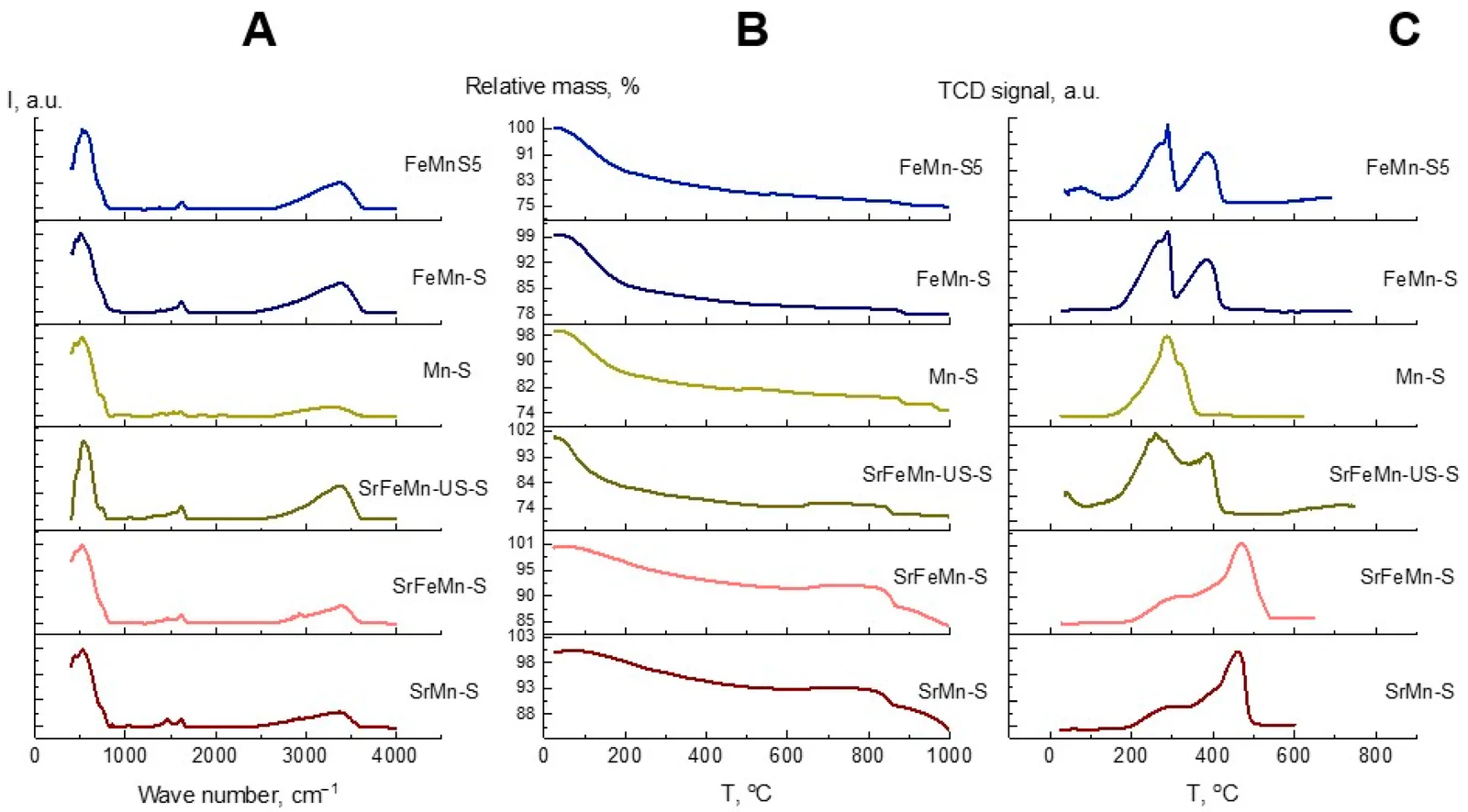
Spectroscopic, thermal, and reduction characteristics of samples. (**A**) FTIR spectra, (**B**) TG curves, and (**C**) H_2_-TPR profiles of SrMn-S, SrFeMn-S, SrFeMn-US-S, Mn-S, FeMn-S, and FeMn-S5. The FTIR and H_2_-TPR curves are vertically shifted for clarity, whereas the TG curves are vertically separated and shown with numerical relative-mass scales.

FTIR, TG/DTG, and H_2_-TPR were used together to evaluate hydration, thermolabile states, and reduction behavior after physical treatment (Figure 4). The FTIR spectra of all samples contain a broad O-H stretching band in the 3300–3400 cm^−1^ region, an H_2_O bending band at 1613–1624 cm^−1^, and an intense low-frequency Mn-O band in the 507–532 cm^−1^ region (Figure 4A, Table S3). These features indicate the presence of hydrated/hydroxylated surface states while preserving the Mn-O framework [35,36,42].

In the Sr-containing series, the *I_OH_*/*I_Mn-O_* ratio increases from 0.192 for SrMn-S to 0.217 for SrFeMn-S, indicating a moderate increase in the relative contribution of O-H-containing states after Fe modification. After ultrasonic treatment, SrFeMn-US-S exhibits the most pronounced hydration response: *I_OH_*/*I_Mn-O_* increases to 0.426, while *I_H_*_/*O*_/*I_Mn-O_* increases to 0.169. At the same time, the Mn-O band position shifts from 524 cm^−1^ for SrFeMn-S to 530 cm^−1^ for SrFeMn-US-S. Taken together, these changes are consistent with a more hydrated Mn-O-H environment and an increased contribution of readily reducible Mn-O states in the recovered SrFeMn-US-S solid after the overall ultrasonic processing/recovery route.

FeMn-S also exhibits a substantially higher *I_OH_*/*I_Mn-O_* value of 0.367. This coincides with its higher N_2_-accessible surface area and pore volume, although the present dataset is insufficient to establish a quantitative relationship between these descriptors. After vibratory milling, FeMn-S5 retains the main O-H/H_2_O and Mn-O bands; however, *I_OH_*/*I_Mn-O_* decreases to 0.329, and *I_H_*_2*O*_/*I_Mn-O_* decreases from 0.138 to 0.081 (Table S3). Thus, milling does not eliminate hydrated surface states but changes their relative contribution against the background of a substantial decrease in N_2_-accessible mesoporosity.

TG/DTG analysis is consistent with the FTIR data and further reveals differences in the amount of thermolabile water- and hydroxyl-containing components. SrMn-S shows the lowest total mass loss, 14.8%, over the 25–1000 °C range, with a mass loss below 120 °C of only 0.14%. For SrFeMn-S, the total mass loss increases to 16.2%, and the low-temperature mass loss increases to 0.87%, consistent with a moderate increase in the contribution of weakly bound water after Fe modification. The most pronounced thermogravimetric hydration response is observed for SrFeMn-US-S: the total mass loss reaches 28.5%, and the mass loss below 120 °C reaches 12.8%. The increased low-temperature mass loss suggests a higher contribution of weakly bound water after ultrasonic treatment, dialysis, and subsequent drying, consistent with the commonly reported attribution of low-temperature mass loss in hydrated oxides to physisorbed and weakly bound water [43].

For FeMn-S and FeMn-S5, the total mass losses are 21.8% and 25.4%, respectively, while the low-temperature mass losses are 6.5% and 7.3%. The more complex DTG profile of FeMn-S5 may reflect redistribution of thermolabile water- and hydroxyl-containing states after mechanical treatment. However, these changes occur against the background of a pronounced decrease in *S_BET_* and *V_tot_*; therefore, they should be interpreted together with the loss of N_2_-accessible mesoporosity.

H_2_-TPR further differentiates the two physical-treatment routes (Figure 4C). SrMn-S and SrFeMn-S are characterized predominantly by high-temperature reduction, with maxima at approximately 461–470 °C. Relative to SrFeMn-S, the SrFeMn-US-S solid recovered after the ultrasonic processing/recovery route shows a pronounced redistribution of the H_2_-TPR profile toward lower temperature: for SrFeMn-US-S, this maximum is located at approximately 260 °C, with an additional contribution at around 386 °C. This represents a larger low-temperature contribution to the mass-normalized H_2_-TPR response and is consistent with a redistribution toward more readily reducible Mn-O states. Such an interpretation is compatible with reported H_2_-TPR behavior of manganese oxides with different structural arrangements [43,44]. The latter interpretation is considered together with the observed differences in N_2_-accessible mesoporosity and hydration-related descriptors.

FeMn-S exhibits a two-step reduction profile with maxima at approximately 288 and 385 °C. The FeMn-S > 355 µm fraction retains a closely similar profile, with T_max_ values of approximately 287 and 383 °C, and shows only a slight decrease in total H_2_ uptake from 0.38 to 0.36 mmol H_2_ g^−1^ (Figure S1 and Table 2). These results indicate that particle-size fractionation alone has only a limited effect on the reduction behavior of FeMn-S. FeMn-S5 likewise retains similar reduction-maxima positions but shows a lower total H_2_ uptake of 0.34 mmol H_2_ g^−1^. Thus, vibratory milling modestly decreases the mass-normalized extent of reduction without producing a marked shift in the characteristic reduction-temperature regime.

Overall, the recovered solid states were route-dependent. SrFeMn-US-S combined higher accessible mesoporosity, stronger hydration-related signatures, and a larger low-temperature contribution to the H_2_-TPR profile, whereas FeMn-S5 retained the reduction-temperature regime of the parent solid but exhibited lower accessible mesoporosity and lower total H_2_ uptake. These results show that textural, hydration, and reduction descriptors must be interpreted jointly rather than as independent indicators of processing-induced activation.

The as-prepared MnO_x_ powders formed large agglomerated particulate structures immediately after addition to water or buffer. Such rapidly sedimenting agglomerates were unsuitable for reproducible suspension-based comparisons because they hindered homogeneous sampling and reliable dilution.

To generate operationally defined dispersed fractions, a controlled probe-ultrasonication and centrifugation protocol was applied [45,46]. Powders were either manually ground or, for FeMn-S5, ball-milled, and then the resulting fine powders were added to sodium citrate solution and sonicated with a horn probe for 4 h. Sodium citrate was introduced primarily as a dispersion stabilizer. Because citrate should not be regarded as chemically inert toward MnO_x_, partial chemical or complexation effects during prolonged citrate-assisted sonication cannot be excluded [47]. After citrate-assisted processing and subsequent washing, aqueous suspensions were obtained. The pronounced decrease in the Sr/Mn ratio of SrFeMn-US-S indicates that the concentrated recovery route was also accompanied by compositional redistribution. Accordingly, SrFeMn-US-S is interpreted as a product of the overall processing/recovery route rather than of ultrasound alone.

**Figure 5:**
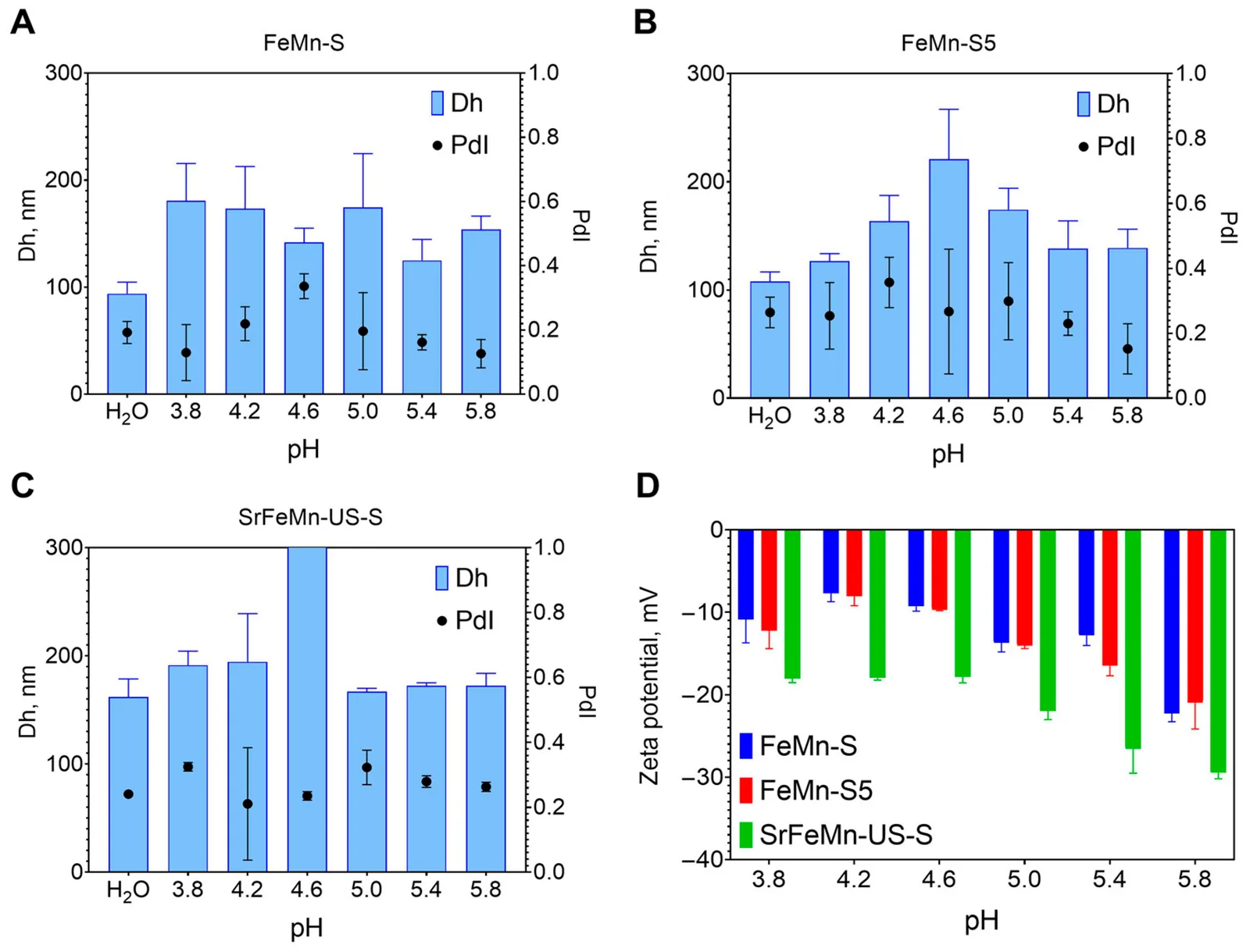
Hydrodynamic diameter (D_h_), polydispersity index (PdI), and ξ-potential of MnO_x_ suspensions. (**A**–**C**) D_h_ and PdI in deionized water and in an assay-mimicking medium without TMB (1 mmol L^−1^ sodium propionate buffer, 10% DMSO); (**D**) ξ-potential in 1 mmol L^−1^ sodium propionate solutions over pH 3.8–5.8. Data are presented as mean ± SD (*n* = 3).

Colloidal stability in deionized water and in the reaction medium (1 mmol L^−1^ sodium propionate buffer with 10% DMSO) was assessed by DLS (Figure 5). During storage in deionized water, the dispersions remained readily redispersible and, after brief sonication, showed hydrodynamic-size values close to their initial values. The measured PdI values (approximately 0.1–0.4, depending on sample and medium) indicate sample-dependent polydispersity of the hydrodynamic size distributions. Because DLS reports an equivalent hydrodynamic diameter, these data do not provide direct information on particle morphology. A low buffer concentration of 1 mmol L^−1^ was used to minimize electrolyte-induced aggregation during the catalytic measurements. Hydrodynamic sizes measured in the buffer/DMSO reaction medium varied by sample: FeMn-S5 and SrFeMn-US-S showed sizes comparable to those in water, whereas FeMn-S exhibited larger sizes in an assay-mimicking buffer/DMSO medium without TMB.

After 10 min incubation in the assay-mimicking medium, most sample/pH combinations did not exhibit markedly larger D_h_ values than those measured in water. FeMn-S showed larger hydrodynamic sizes in the assay-mimicking medium, whereas SrFeMn-US-S exhibited pronounced aggregation at pH 4.6. The ξ-potential remained negative for all three MnO_x_ suspensions throughout the investigated pH range (Figure 5D). SrFeMn-US-S generally exhibited a more negative ξ-potential than FeMn-S and FeMn-S5, whereas the two FeMn samples showed broadly similar values. No simple correspondence was observed between ξ-potential magnitude and D_h_. Thus, differences in surface charge alone do not account for the observed sample- and pH-dependent changes in hydrodynamic size.

The composition of the stable suspension fraction was used as an operational indicator of powder transfer into the dispersed state after ultrasonic treatment. This descriptor was introduced because nanozyme activity measurements are sensitive to colloidal state, aggregation, and stability of the dispersed fraction [3,26,27]. This analysis was performed for the FeMn-S/FeMn-S5 pair, because in this case vibratory milling preceded the identical ultrasonic dispersion step and could therefore affect the amount of material transferred into the liquid fraction.

After identical ultrasonic dispersion, the Mn concentration in the operationally defined stable suspension fraction decreased from 50.5 mg L^−1^ for FeMn-S to 17.3 mg L^−1^ for FeMn-S5, while the corresponding Fe concentration decreased from 10.2 to 3.9 mg L^−1^. Because the powder Fe/Mn ratios remained close, these differences are interpreted as evidence of less efficient transfer of the milled material into the stable dispersed fraction rather than of a change in bulk Fe/Mn composition. One possible explanation is that milling promoted the formation of more compact or re-agglomerated particle assemblies that were less efficiently redispersed and were preferentially removed during the subsequent centrifugation steps. This interpretation is consistent with the pronounced loss of *S_BET_* and *V_tot_*, although the removed sedimented fraction was not directly characterized morphologically. Thus, preliminary milling affected both the dry-state texture and the subsequent powder-to-suspension transfer of FeMn-S.

To determine whether the processing-dependent changes observed in the solid and suspension states were reflected in functional performance, the Mn-normalized chromogenic response was evaluated using TMB as a model substrate [1,3,48]. Michaelis–Menten analysis was not applied because the A_652_ time profiles showed rapid signal development and an early approach to a plateau, precluding robust identification of a common initial linear-rate interval across the analyzed suspensions (Figure S2). Accordingly, the TMB-concentration experiment was evaluated in terms of the concentration dependence of the operational cumulative chromogenic response. ΣA_652_ is interpreted as an empirical within-study response index rather than as a kinetic parameter and should not be directly compared with the Michaelis–Menten constant (*K_m_*), maximum reaction velocity (*V_max_*), initial-rate, or specific-activity values reported for other nanozyme systems. All assays were performed at the same final manganese concentration (500 ng Mn mL^−1^; 9.1 µmol Mn L^−1^). Chromogenic-response comparisons were restricted to the Fe-containing samples for which stable suspension fractions were prepared and compositionally characterized; Mn-S and SrMn-S were retained as dry-state references and were not included in the activity analysis.

**Figure 6:**
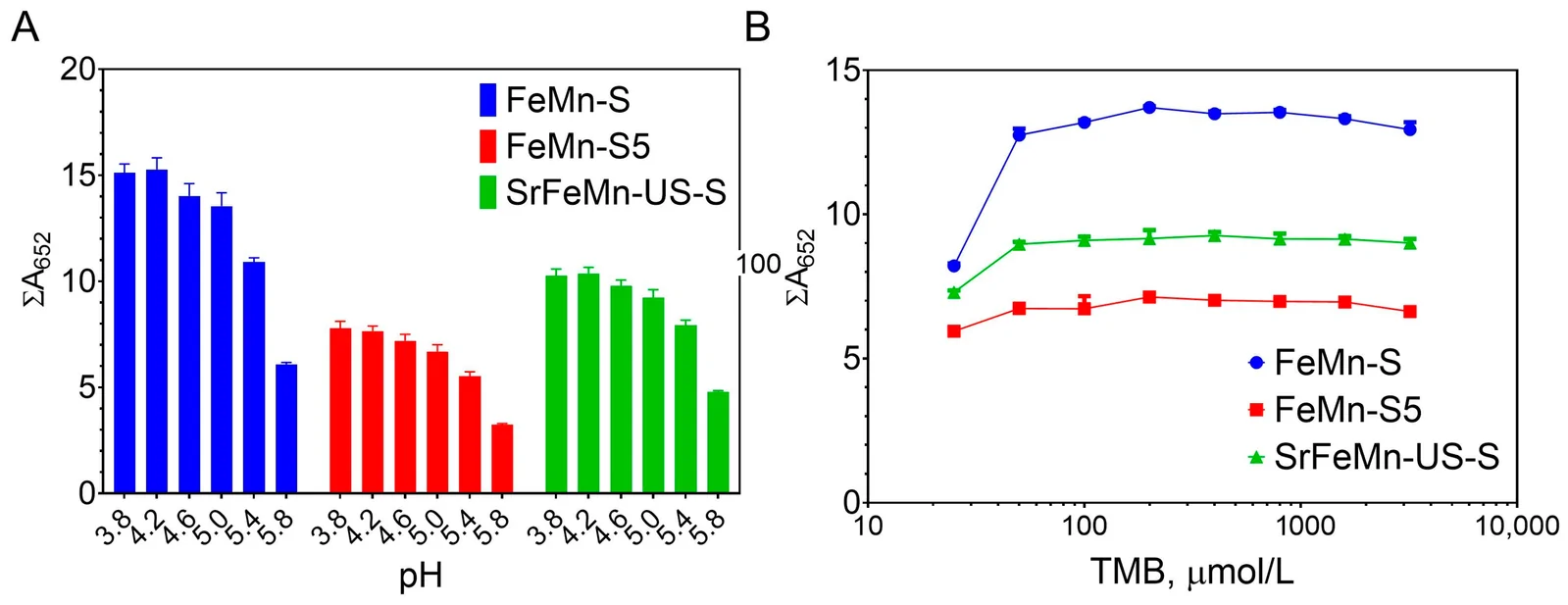
Mn-normalized cumulative A_652_ response (ΣA_652_) of MnO_x_ nanozyme suspensions. (**A**) Effect of pH at a fixed TMB concentration of 0.4 mmol L^−1^. (**B**) Effect of TMB concentration at pH 3.8. The final Mn concentration was 500 ng Mn mL^−1^ in all assays. Data are presented as mean ± SD (*n* = 3).

The highest ΣA_652_ responses were observed at pH 3.8 and 4.2 for all tested suspensions (Figure 6A). pH 3.8 was selected for the subsequent TMB-concentration experiments because it produced one of the highest ΣA_652_ responses among the tested conditions. The ΣA_652_ response increased most strongly between 25 and approximately 100 µmol L^−1^ TMB and subsequently changed only moderately over the higher concentration range, particularly for FeMn-S and SrFeMn-US-S.

The FeMn-S/FeMn-S5 pair revealed two distinct consequences of vibratory milling. First, milling decreased the amount of Fe-MnO_x_ material recovered in the stable suspension fraction, as indicated by the approximately threefold lower Mn concentration after identical ultrasonic dispersion. Second, after adjustment of both suspensions to the same final Mn concentration of 500 ng Mn mL^−1^, FeMn-S5 still exhibited a lower ΣA_652_ response than FeMn-S. Thus, the difference in chromogenic response cannot be attributed to unequal Mn loading in the assay. Milling therefore affected two distinct material-level outcomes: the amount of Fe-MnO_x_ transferred into the stable suspension fraction and the functional response per unit Mn within that dispersed fraction.

The Sr-containing series provides a complementary route-specific observation rather than a direct quantitative counterpart to the FeMn-S/FeMn-S5 comparison. The SrFeMn-US-S solid recovered through the concentrated processing route exhibits higher N_2_-accessible surface area, stronger hydration-related features, and a larger low-temperature contribution to the H_2_-TPR profile than SrFeMn-S. In the related working-suspension route, however, the Mn-normalized ΣA_652_ response remains lower than that of FeMn-S. Because SrFeMn-US-S was obtained by a concentrated processing/recovery protocol rather than by drying the actual assay suspension, its powder-state descriptors are interpreted as route-associated characteristics and not as direct quantitative predictors of the measured ΣA_652_ response. These observations therefore indicate that increased N_2_-accessible mesoporosity of a recovered solid alone is insufficient to predict the ordering of the Mn-normalized chromogenic response.

The term ‘suspension-accessible MnO_x_ state’ is used here as an operational framework for the fraction of the material present in a dispersed form on the assay-relevant time scale and capable of contributing to the Mn-normalized chromogenic response. This state was not represented by a directly measured interfacial area and could not be assigned to a single structural descriptor. Instead, it reflected the combined consequences of processing history, accessible mesoporosity, hydration, reducibility, and powder-to-suspension transfer.

## 4. Conclusions

Physical processing produced distinct route-dependent changes across the powder-to-suspension sequence of the studied nanostructured MnO_x_ materials. The concentrated SrFeMn processing/recovery route yielded a Sr-depleted recovered solid with increased N_2_-accessible surface area and pore volume, stronger hydration-related signatures, and a greater low-temperature contribution to the H_2_-TPR profile; these changes are attributed to the complete processing/recovery sequence rather than to ultrasound alone. In the FeMn-S/FeMn-S5 pair, vibratory milling preserved the bulk Fe/Mn ratio, markedly decreased accessible mesoporosity and transfer into the stable suspension fraction, and also lowered H_2_ uptake and the Mn-normalized time-summed ΣA_652_ response.

These contrasting outcomes demonstrate that solid-state properties, powder-to-suspension transfer, and functional response represent distinct but interconnected processing-dependent characteristics. Because these descriptors correspond to different operational states of the material, the present dataset does not support identification of a single quantitative solid-state predictor of suspension-phase functionality; the observed associations should therefore be interpreted as route-dependent rather than as formal correlations. For powder-derived MnO_x_ materials, processing history should consequently be considered part of the material description and reported together with solid-state and suspension-state characterization.

## Figures and Tables

**Table 1:** Elemental composition and molar ratios of MnO_x_ powders and stable suspension fractions.

**Sample**	**State**	**Mn, wt%**	**Fe, wt%**	**Sr, wt%**	**Fe/Mn, mol mol^−1^**	**Sr/Mn, mol mol^−1^**
SrMn-S	Powder	25.83	n.d.	15.51	n.d.	0.3765
SrFeMn-S	Powder	23.75	1.68	12.65	0.0694	0.3339
SrFeMn-US-S	Powder	28.03	1.75	5.83	0.0615	0.1304
Mn-S	Powder	32.89	n.d.	0.03	n.d.	0.0006
FeMn-S	Powder	28.62	5.03	n.d.	0.1728	n.d.
FeMn-S5	Powder	28.90	5.22	n.d.	0.1777	n.d.
**Sample**	**State**	**Mn, mg L^−1^**	**Fe, mg L^−1^**	**Sr, mg L^−1^**	**Fe/Mn, mol mol^−1^**	**Sr/Mn, mol mol^−1^**
SrMn-S	Stable suspension	30.806	n.d.	7.331	n.d.	0.1492
SrFeMn-S	Stable suspension	13.988	2.103	2.405	0.1479	0.1078
FeMn-S	Stable suspension	50.454	10.156	n.d.	0.1980	n.d.
FeMn-S5	Stable suspension	17.316	3.917	n.d.	0.2226	n.d.

Note: n.d.—not detected. Powder compositions are expressed as wt% calculated from acid dissolution of 10 mg of powder followed by dilution to 50 mL. Suspension compositions are expressed as concentrations recalculated to the initial suspension before dilution.

**Table 2:** Textural, thermal, and reduction parameters of the samples.

Sample	*S_BET_*, m^2^ g^−1^	*V_tot_*, cm^3^ g^−1^	*d_av_*, nm	Total Mass Loss, %	T_max_, °C	H_2_ Uptake, mmol g^−1^
SrMn-S	131.0	0.165	5.0	14.8	461	0.28
SrFeMn-S	150.8	0.168	4.5	16.2	470	0.33
SrFeMn-US-S	241.5	0.277	4.6	28.5	260, 386	0.36
Mn-S	237.0	0.238	4.0	25.2	287	0.43
FeMn-S	305.9	0.533	7.0	21.8	288, 385	0.38
FeMn-S > 355 µm	291.2	0.504	6.9	n.d.	287, 383	0.36
FeMn-S5	127.1	0.215	6.8	25.4	288, 386	0.34

Note: n.d.—not determined.

## Data Availability

The original contributions presented in this study are included in the article/Supplementary Materials. Further inquiries can be directed to the corresponding author.

## Supplementary Materials

The following supporting information can be downloaded at: https://www.mdpi.com/article/10.3390/nano16181141/s1, Table S1: Representative literature survey of powder-to-suspension preparation and colloidal-state reporting in oxide, mixed-oxide, and related nanozyme assays; Table S2: XRD peak parameters and apparent coherence lengths, and candidate PDF-2 matches of the MnO_x_ materials; Table S3: Positions and relative intensities of the O-H, H_2_O, and Mn-O bands in the FTIR spectra of samples; Figure S1: H_2_-TPR profiles of FeMn-S and the FeMn-S > 355 μm fraction; Figure S2: Representative A_652_ vs. reaction time at 0.4 mmol L^−1^ TMB, pH 3.8. References [3,5,14,15,16,17,18,19,20,25,26,27,49,50,51,52,53,54,55,56,57,58,59,60,61,62,63,64,65,66,67,68,69,70,71,72,73,74,75,76,77,78,79,80] are cited in the Supplementary Materials.
