# Supplementary material for: Physical Processing Controls the Structure, Mesoporosity, and Suspension-Phase Functionality of Nanostructured MnOx Materials
Source: Nanomaterials (Basel). 2026 Sep 11;16(18):1141. doi: 10.3390/nano16181141 (PMC13610552; doi:10.3390/nano16181141)
Supplement: Supplementary file 1 [file nanomaterials-16-01141-s001.zip › nanomaterials-4542474-supplementary.pdf]

Supplementary Materials

Physical Processing Controls the Structure, Mesoporosity, and Suspension-Phase Functionality of Nanostructured MnO_x Materials

Ekaterina Saenko[a]*, Pavel Khramtsov[b], Igor Valtsifer[a], Anastasia Novokshonova[b], Viktor Valtsifer[a]

[a] *Institute of Technical Chemistry, Ural Branch of the Russian Academy of Sciences, Branch of the Perm Federal Research Center, 3 Akademika Koroleva St., Perm 614068, Russian Federation*

[b] *Institute of Ecology and Genetics of Microorganisms, Ural Branch of the Russian Academy of Sciences, Branch of the Perm Federal Research Center, 13 Goleva St., Perm 614081, Russian Federation*

* Corresponding author: saenko.e@itcras.ru

Keywords: nanostructured manganese oxides; MnO_x; mesoporous nanomaterials; physical processing; structure-property relationships; colloidal dispersion; nanozyme

Contents:

- Table S1. Representative literature survey of powder-to-suspension preparation and colloidal-state reporting in oxide, mixed-oxide, and related nanozyme assays.
- Table S2. XRD peak parameters and apparent coherence lengths, and candidate PDF-2 matches of the MnO_x materials.
- Table S3. Positions and relative intensities of the O-H, H_2O, and Mn-O bands in the FTIR spectra of samples.
- Figure S1. H_2-TPR profiles of FeMn-S and the FeMn-S >355 μm fraction.
- Figure S2. Representative A_{652} vs. reaction time at 0.4 mmol L^{-1} TMB, pH 3.8.
- References.

The studies included in Table S1 were selected to represent oxide, mixed-oxide, and related heterogeneous nanozyme systems commonly used in suspension-based activity assays. The survey provides a qualitative assessment of the completeness of powder-to-suspension preparation and colloidal-state reporting. It is illustrative rather than exhaustive and was not intended as a systematic review, bibliometric analysis, or estimate of field-wide prevalence. The classification is based solely on information explicitly reported in the articles and their Supplementary Information.

Table S1. Representative literature survey of powder-to-suspension preparation and colloidal-state reporting in oxide, mixed-oxide, and related nanozyme assays.

No	Material/ nanozyme	Thermal treatment/ calcination	Activity type	Reported preparation of catalytic suspension	Colloidal stability data	Operational reporting category	DOI/Ref.
1	Fe_2O_3 mesoporous iron oxide nanozyme	Several steps, 523-623 K	POX-like	Not described in operational detail	Not available	Limited reporting	10.1039/C9TB00989B [1]
2	Co_3O_4@Co-Fe oxide double-shelled nanocages	350 °C, 2 h	POX-like; OX-like	Not described in operational detail	Not available	Limited reporting	10.1016/j.cej.2020.125130 [2]
3	Fe_3O_4/mesoporous graphitized carbon composite	450 °C, 50 min	POX-like	Not described in operational detail	Not available	Limited reporting	10.1007/s00604-021-05035-1 [3]
4	Iron oxide nanozyme	250-450 °C, 2 h	POX-like	Not described in operational detail	Not available	Limited reporting	10.1002/cnma.201800487 [4]
5	CoO_x nanozyme	Twice at 500 °C, 2 h	POX-like	Not described in operational detail	Not available	Limited reporting	10.1007/s00216-020-02815-1 [5]
6	Co_3O_4 nanozyme	450 °C, 2 h	POX-like	Not described in operational detail	Not available	Limited reporting	10.1021/acs.jafc.0c08208 [6]
7	$Co_3V_2O_8/Co_3O_4$ mixed oxide	300-800 °C, 2 h	POX-like; OX-like	Not described in operational detail	Not available	Limited reporting	10.1016/j.apcatb.2019.118256 [7]
8	Mesoporous CuO hollow sphere nanozyme	350 °C, 2 h	POX-like	Assay composition reported; dispersion protocol not fully specified	Not available	Partial reporting	10.3390/bios11080258 [8]

9	Co-Al-Ce mixed metal oxides	200-800 °C, 2 h	POX-like	Not described in operational detail	Not available	Limited reporting	10.1007/s00343-020-0041-6 [9]
10	$NiCo_2O_4$ nanozyme	300 °C, 5 h	POX-like; OX-like	Not described in operational detail	Not available	Limited reporting	10.1016/j.jhazmat.2020.123939 [10]
11	$PrO_{1.8}$ nanozyme	800 °C, 4 h	POX-like	Suspension preparation not separated as a controlled catalytic variable	Available/partial	Partial reporting	10.1038/s41598-020-61452-x [11]
12	$MnFe_2O_4$/C MOF-derived bimetallic nanozyme	450 °C, 2 h	CAT-like	Not described in sufficient detail for catalytic dispersion state	Available/partial	Partial reporting	10.1016/j.matdes.2021.109646 [12]
13	Perovskite and transition-metal oxides	Multistep treatment, 180-850 °C	POX-like	Nanozyme concentration and assay conditions reported; dispersion protocol only generally described	Not available	Partial reporting	10.1038/s41467-019-08657-5 [13]
14	Ultrathin porous Co_3O_4 nanosheets	450 °C, 2 h	POX-like	Nanozyme assay reported; complete dispersion procedure and reaction-medium stability not	Available / partial	Partial reporting	10.1007/s12274-021-3656-9 [14]

				reported as core variables			
15	Fe-doped SiO_2 nanozyme	550 °C, 6 h	POX-like	Not described in operational detail	Not available	Limited reporting	10.1016/j.colsurfb.2022.112603 [15]
16	$Ca_2Mn_8O_{16}$ nanozyme	400 °C, 3 h	CAT-like; OX-like; POX-like	Sonication mentioned; operational parameters not fully reported	Partial; water/PBS only	Partial reporting	10.1002/adma.202312316 [16]
17	Mn_3O_4 nanozyme	300 °C, 5 h	OX-like; POX-like	Not described in operational detail	Available	Partial reporting	10.1016/j.micromeso.2022.111826 [17]
18	NiO nanozyme	400 °C, 12 h	OX-like; POX-like	Not described in operational detail	Not available	Limited reporting	10.3390/nano11051285 [18]
19	Co_3O_4/MO_3, M = Mo or W	450 °C, 2 h	OX-like; POX-like	Described in general terms; colloidal state in reaction medium not fully characterized	Not available	Partial reporting	10.1039/D0TB01337D [19]
20	ZnO-Co_3O_4 nanozyme	350 °C, 3 h	OX-like; POX-like	Not described in operational detail	Not available	Limited reporting	10.1007/s00604-021-04705-4 [20]
21	NiO nanozyme	600 °C, 5 h	POX-like	Not described in operational detail	Not available	Limited reporting	10.1039/D4NA00932K [21]
22	P_2W_{18}@Fe_2O_3 nanozyme	400 °C, 2 h	POX-like	Not described in operational detail	Not available	Limited reporting	10.1007/s10904-024-03192-3 [22]
23	CaO nanozyme	800 °C, 1 h	POX-like	Not described in operational detail	Not available	Limited reporting	10.1007/s10904-023-02906-3 [23]

24	Pt/Al_2O_3 nanozyme	300-600 °C, 2 h	POX-like	Not described in operational detail	Not available	Limited reporting	10.1002/cplu.202300609 [24]
25	Au-iron oxide nanozyme	800 °C, 8 h	POX-like	Not described in operational detail	Not available	Limited reporting	10.1002/asia.202401479 [25]
26	CeO_x/Mn_3O_4 nanozyme	400 °C, 12 h	CAT-like; SOD-like	Not described in operational detail	Not available	Limited reporting	10.1002/anie.202416686 [26]
27	CoMnFe layered double oxides	250 °C, 5 h	CAT-like; POX-like	Not described in operational detail	Partial; water, DMEM and PBS only	Partial reporting	10.1002/advs.202403107 [27]
28	Spinel oxide nanozymes, including $CuCo_2O_4$ and related spinels	300-800 °C depending on oxide	POX-like	Nanozyme concentration and assay reported; dispersion/stability not the main reported descriptor	Not available / not central	Partial reporting	10.1002/anie.202421790 [28]
29	MnO_x@NC nanozyme	500 °C, 2-8 h	OX-like; POX-like	Not described in operational detail	Not available	Limited reporting	10.1002/cjoc.202400938 [29]
30	Fe_3O_4@Co_3O_4/CN nanozyme	800 °C, 3 h	OX-like; POX-like	Not described in operational detail	Not available	Limited reporting	10.3390/chemosensors12060104 [30]
31	Fe_3O_4/CN nanozyme	300-500 °C, 1 h	OX-like	Not described in operational detail	Not available	Limited reporting	10.1002/smll.202408609 [31]
32	TiO_2@CeO_x	645 °C, 8 h	POX-like	Not described in operational detail	Not available	Limited reporting	10.1021/am5057129 [32]
33	$LaCoO_3$ nanozyme	Gel treatment followed by 400	POX-like; CAT-like	Nanozyme concentration, buffer and	Not available	Partial reporting	10.1039/C7NJ01177F [33]

		and 700 °C calcination		reaction volume reported; no complete colloidal stability control			
34	$LaNiO_3$ nanocubes and reduced/reoxidized derivatives	650-700 °C; reduction at 350 °C	POX-like	Stock suspension concentration and assay volumes reported; detailed dispersion protocol/stability not fully controlled	Not available	Partial reporting	10.7150/thno.19257 [34]
35	$NiCo_2O_4$ hierarchical self-assembled nanozyme	300 °C, 2 h	POX-like; antibacterial ROS generation	Suspension concentration range reported; complete colloidal stability in reaction medium not central	Not available/incomplete	Partial reporting	10.1002/adma.202210455 [35]
36	Fe_3O_4, γ-Fe_2O_3, α-Fe_2O_3 and $LiFePO_4$ nanoparticles	200 and 650 °C, 2 h	POX-like	Ultrasonic dispersion reported	Hydrodynamic diameter and ξ-potential reported	Detailed reporting	10.1038/s41467-022-33098-y [36]
37	CeO_2 nanozyme	300 and 500 °C, 2 h	Photolyase-like/oxidoreductase-related	Not described in operational detail	Not available	Limited reporting	10.1021/acs.nanolett.9b03836 [37]
38	MnO and Mn_3O_4 nanozymes	MnO: 593 K under Ar, 30 min; Mn_3O_4: 573 K, 4 h	SOD-like; CAT-like	Ligand exchange reported;	Not available	Partial reporting	10.1039/C6TB02078J [38]

				catalytic dispersion stability not fully reported as a reaction-medium descriptor			
39	Commercial manganese oxides: MnO, MnO_2, Mn_2O_3, Mn_3O_4	Commercial/synthesis-dependent	OX-like; POX-like	Preparation described partly; DLS/TEM reported for some systems	DLS/TEM available	Detailed reporting	10.1039/D3NR05127G [39]
40	Fe_3O_4 and $CoFe_2O_4$ nanozymes for nanozyme-strip	Not central/size-controlled oxide nanozymes	POX-like; TMB/H_2O_2	Ultrasonication time before DLS/ξ-potential and activity measurement reported	PDI and ξ-potential reported	Detailed reporting	10.3390/s23073414 [40]
41	MnO_x/C nanozymes with valence-dependent activity	Thermal conversion / MnO_x formation	POX-like; TMB/H_2O_2	Ultrasonic dispersion to stock suspension reported; sonication power/time not fully specified	Not reported for reaction medium	Partial reporting	10.1038/s41598-025-26164-0 [41]
42	$CoFe_2O_4$/GO@Dex-CS nanozyme	Ferrite-based composite	POX-like; TMB/H_2O_2	Sonication in acetate buffer reported; full colloidal stability in reaction medium not	Not fully available	Partial reporting	10.1038/s41598-026-43315-z [42]

				fully established			
43	Prussian Blue nanozymes	Not oxide; colloidal benchmark	HRP-like/POX-like	Colloidal stability explicitly investigated as a function of electrolyte-induced aggregation	Aggregation and activity analysed	Detailed reporting	10.1021/acs.jpcb.5c01256 [43]
44	Oxidoreductase-mimicking nanozymes	Methodological study	OX-like; POX-like and related oxidoreductase activities	Colloidal stability, aggregation and chemical transformations explicitly discussed as factors affecting kinetic interpretation	Available/directly discussed	Detailed reporting	10.1021/acsnano.4c12539 [44]

Note: POX-like – peroxidase-like activity; OX-like – oxidase-like activity; CAT-like – catalase-like activity; SOD-like – superoxide dismutase-like activity; HRP-like – horseradish peroxidase-like activity; TMB – 3,3',5,5'-tetramethylbenzidine; PDI – polydispersity index; DLS – dynamic light scattering. The categories refer only to the completeness of explicitly reported dispersion and colloidal-state information and do not represent an assessment of scientific quality, methodological rigor, or overall significance of the cited studies.

Table S2. XRD peak parameters and apparent coherence lengths, and candidate PDF-2 matches of the MnO_x materials.

Sample	2θ (37),°	FWHM (37),°	D_{app} (37), nm	2θ (66),°	FWHM (66),°	D_{app} (66), nm	Candidate PDF-2 Search/Match entries
SrMn-S	37.13	2.05	4.1	66.22	2.41	3.9	MnO_2: 00-030-0820, 00-053-0633, 00-042-1316; $Mn(OH)_4$: 00-015-0604; $Mn(O,OH)_2$: 00-017-0510; $Mn^{3+}O(OH)$: 00-012-0733
SrFeMn-S	37.08	2.01	4.2	66.03	2.65	3.6	MnO_2: 00-042-1169, 00-043-1455, 00-044-0141; $Mn(OH)_4$: 00-015-0604; $Mn^{3+}O(OH)$: 00-018-0804
SrFeMn-US-S	37.28	2.11	4.0	66.22	2.43	3.9	MnO_2: 00-044-0992, 00-042-1169; $Fe(OH)_2$: 01-073-6991, 00-013-0089; $Mn^{3+}O(OH)$: 00-018-0804; $Sr_2Mn_{14}O_{27}\cdot xH_2O$: 00-050-0012; $Mn_7O_{13}\cdot 5H_2O$: 00-023-1239; $Mn(OH)_4$: 00-015-0604; MnO: 00-007-0230
Mn-S	37.20	1.83	4.6	66.33	2.97	3.2	MnO: 00-007-0230; MnO_2: 00-053-0633; $Mn(OH)_4$: 00-015-0604; $Mn(OH)_2$: 00-018-0787
FeMn-S	37.07	2.36	3.6	66.18	3.43	2.8	$Mn(OH)_4$: 00-015-0604; MnO_2: 00-053-0633, 00-042-1316; $Mn^{3+}O(OH)$: 00-024-0713; $Mn(OH)_2$: 00-018-0787
FeMn-S5	37.02	2.33	3.6	66.16	3.57	2.7	$Mn(OH)_4$: 00-015-0604; MnO_2: 00-053-0633, 00-042-1316; $Mn^{3+}O(OH)$: 00-024-0713; $Mn(OH)_2$: 00-018-0787

Note. D_{app} was calculated independently from the broad diffraction features near 37° and 66° 2θ using the Scherrer equation. Instrumental broadening was not corrected, and the values obtained from the two features were not averaged. PDF-2 entries represent candidate Search/Match matches only.

Table S3. Positions and relative intensities of the O-H, H_2O, and Mn-O bands in the FTIR spectra of samples.

Sample	ν_{OH}, cm^{-1}	I_{OH}	ν_{H2O}, cm^{-1}	I_{H2O}	ν_{Mn-O}, cm^{-1}	I_{Mn-O}	I_{H2O}/I_{Mn-O}	I_{OH}/I_{Mn-O}
SrMn-S	3374	0.134	1616	0.070	522	0.700	0.100	0.192
SrFeMn-S	3394	0.062	1618	0.031	524	0.287	0.107	0.217
SrFeMn-US-S	3383	0.334	1621	0.133	530	0.784	0.169	0.426
Mn-S	3293	0.155	1613	0.066	517	1.430	0.046	0.108
FeMn-S	3383	1.016	1622	0.381	507	2.766	0.138	0.367
FeMn-S5	3379	0.957	1624	0.235	521	2.912	0.081	0.329

Note. The intensities I_{OH}, I_{H2O} and I_{Mn-O} were determined as the maximum band intensities in the 3000-3700, 1500-1700, and 450-650 cm^{-1} regions, respectively. The I_{H2O}/I_{Mn-O} and I_{OH}/I_{Mn-O} ratios were used as semi-quantitative indicators of the relative contribution of hydrated/hydroxylated states.

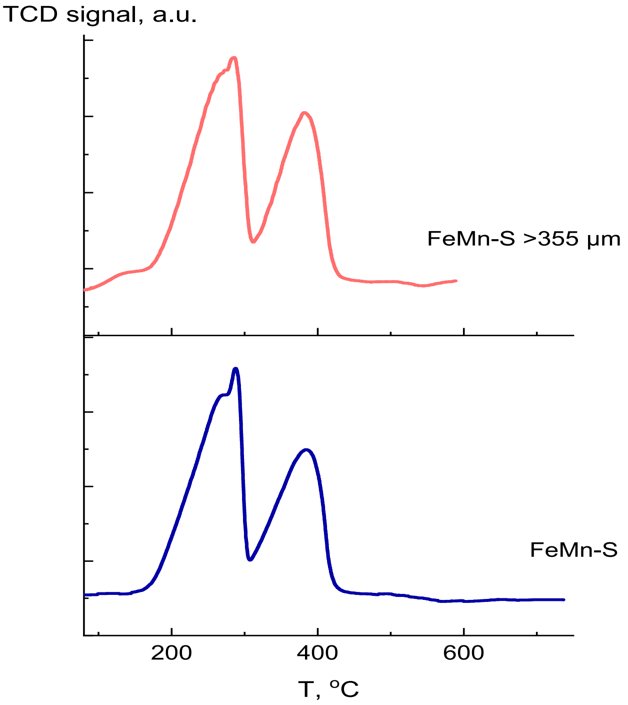

Figure S1. H_2-TPR profiles of FeMn-S and the FeMn-S >355 µm fraction. The profiles are vertically shifted for clarity. Both samples show closely similar two-step reduction behavior with maxima at approximately 288/385 °C for FeMn-S and 287/383 °C for FeMn-S >355 µm. The near coincidence of the reduction maxima indicates closely similar characteristic reduction-temperature regimes, whereas the moderately lower total H_2 uptake of the >355 µm fraction indicates a limited decrease in the mass-normalized extent of reduction under the H_2-TPR conditions.

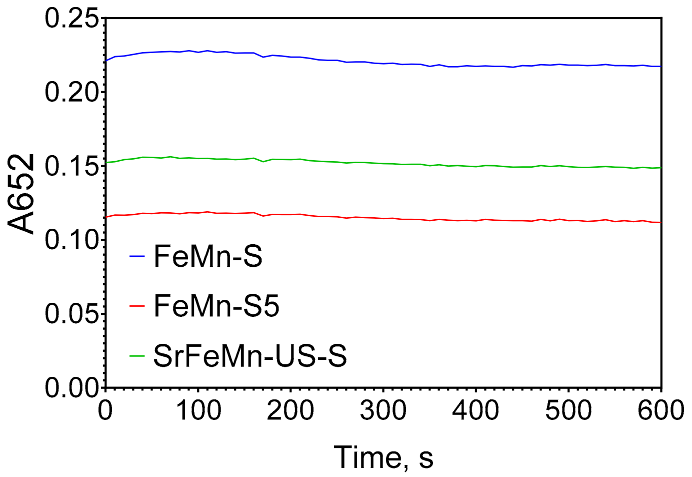

Figure S2. Representative A_{652} vs. reaction time at 0.4 mmol L^{-1} TMB, pH 3.8. Mean values are shown.

References

1. Masud, M.K.; Kim, J.; Billah, Md.M.; Wood, K.; Shiddiky, M.J.A.; Nguyen, N.-T.; Parsapur, R.K.; Kaneti, Y.V.; Alshehri, A.A.; Alghamidi, Y.G.; et al. Nanoarchitectured Peroxidase-Mimetic Nanozymes: Mesoporous Nanocrystalline α- or γ-Iron Oxide? *J. Mater. Chem. B* **2019**, *7*, 5412–5422, doi:10.1039/C9TB00989B.

2. Chen, Q.; Zhang, X.; Li, S.; Tan, J.; Xu, C.; Huang, Y. MOF-Derived Co_3O_4@Co-Fe Oxide Double-Shelled Nanocages as Multi-Functional Specific Peroxidase-like Nanozyme Catalysts for Chemo/Biosensing and Dye Degradation. *Chem. Eng. J.* **2020**, *395*, 125130, doi:10.1016/j.cej.2020.125130.

3. Xu, Z.; Li, L.; Li, K.; Chen, M.-L.; Tu, J.; Chen, W.; Zhu, S.-H.; Cheng, Y.-H. Peroxidase-Mimetic Activity of a Nanozyme with Uniformly Dispersed Fe_3O_4 NPs Supported by Mesoporous Graphitized Carbon for Determination of Glucose. *Microchim. Acta* **2021**, *188*, 421, doi:10.1007/s00604-021-05035-1.

4. Tanaka, S.; Masud, M.K.; Kaneti, Y.V.; Shiddiky, M.J.A.; Fatehmulla, A.; Aldhafiri, A.M.; Farooq, W.A.; Bando, Y.; Hossain, Md.S.A.; Yamauchi, Y. Enhanced Peroxidase Mimetic Activity of Porous Iron Oxide Nanoflakes. *ChemNanoMat* **2019**, *5*, 506–513, doi:10.1002/cnma.201800487.

5. Song, H.; Niu, X.; Ye, K.; Wang, L.; Xu, Y.; Peng, Y. A Novel Alkaline Phosphatase Activity Sensing Strategy Combining Enhanced Peroxidase-Mimetic Feature of Sulfuration-Engineered CoO_x with Electrostatic Aggregation. *Anal. Bioanal. Chem.* **2020**, *412*, 5551–5561, doi:10.1007/s00216-020-02815-1.

6. Luo, D.; Huang, X.; Liu, B.; Zou, W.; Wu, Y. Facile Colorimetric Nanozyme Sheet for the Rapid Detection of Glyphosate in Agricultural Products Based on Inhibiting Peroxidase-Like Catalytic Activity of Porous $Co_3 O_4$ Nanoplates. *J. Agric. Food Chem.* **2021**, *69*, 3537–3547, doi:10.1021/acs.jafc.0c08208.

7. Wang, Y.; Chen, C.; Zhang, D.; Wang, J. Bifunctionalized Novel Co-V MMO Nanowires: Intrinsic Oxidase and Peroxidase like Catalytic Activities for Antibacterial Application. *Appl. Catal. B Environ.* **2020**, *261*, 118256, doi:10.1016/j.apcatb.2019.118256.

8. Cheng, D.; Qin, J.; Feng, Y.; Wei, J. Synthesis of Mesoporous CuO Hollow Sphere Nanozyme for Paper-Based Hydrogen Peroxide Sensor. *Biosensors* **2021**, *11*, 258, doi:10.3390/bios11080258.

9. Chen, C.; Wang, Y.; Zhang, D. Bifunctional Nanozyme Activities of Layered Double Hydroxide Derived Co-Al-Ce Mixed Metal Oxides for Antibacterial Application. *J. Oceanol. Limnol.* **2020**, *38*, 1233–1245, doi:10.1007/s00343-020-0041-6.

10. Mu, Q.; Sun, Y.; Guo, A.; Xu, X.; Qin, B.; Cai, A. A Bifunctionalized $NiCo_2O_4$-Au Composite: Intrinsic Peroxidase and Oxidase Catalytic Activities for Killing Bacteria and Disinfecting Wound. *J. Hazard. Mater.* **2021**, *402*, 123939, doi:10.1016/j.jhazmat.2020.123939.

11. Wang, L.; Liu, Y.; Lu, C.; Yang, Z.; Liu, Y.; Wang, Y.; Rao, H.; Zhang, W.; Wang, X. Ultrasonic Synthesis of Nano-$PrO_{1.8}$ as Nanozyme for Colorimetric Determination of Trans-Resveratrol. *Sci. Rep.* **2020**, *10*, 4432, doi:10.1038/s41598-020-61452-x.

12. Han, X.; Li, Y.; Zhou, Y.; Song, Z.; Deng, Y.; Qin, J.; Jiang, Z. Metal-Organic Frameworks-Derived Bimetallic Nanozyme Platform Enhances Cytotoxic Effect of Photodynamic Therapy in Hypoxic Cancer Cells. *Mater. Des.* **2021**, *204*, 109646, doi:10.1016/j.matdes.2021.109646.

13. Wang, X.; Gao, X.J.; Qin, L.; Wang, C.; Song, L.; Zhou, Y.-N.; Zhu, G.; Cao, W.; Lin, S.; Zhou, L.; et al. Eg Occupancy as an Effective Descriptor for the Catalytic Activity of Perovskite Oxide-Based Peroxidase Mimics. *Nat. Commun.* **2019**, *10*, 704, doi:10.1038/s41467-019-08657-5.

14. Lu, W.; Yuan, M.; Chen, J.; Zhang, J.; Kong, L.; Feng, Z.; Ma, X.; Su, J.; Zhan, J. Synergistic Lewis Acid-Base Sites of Ultrathin Porous Co_3O_4 Nanosheets with Enhanced Peroxidase-like Activity. *Nano Res.* **2021**, *14*, 3514–3522, doi:10.1007/s12274-021-3656-9.

15. Fan, X.; Gong, X.; Zhou, F.; Chen, B.; Tan, S.; Xu, H.; Pan, A.; Liang, S.; He, Y. Mesoporous Peroxidase Nanozyme for Synergistic Chemodynamic Therapy and

Chemotherapy. *Colloids Surf. B Biointerfaces* **2022**, *216*, 112603, doi:10.1016/j.colsurfb.2022.112603.

16. Wang, Z.; Wang, X.; Dai, X.; Xu, T.; Qian, X.; Chang, M.; Chen, Y. 2D Catalytic Nanozyme Enables Cascade Enzyodynamic Effect-Boosted and Ca^{2+} Overload-Induced Synergistic Ferroptosis/Apoptosis in Tumor. *Adv. Mater.* **2024**, *36*, 2312316, doi:10.1002/adma.202312316.

17. Zeng, X.; Yan, S.; Liu, B.-F. Active Nanozyme Derived from Biomineralized Metal-Organic Frameworks for Cholesterol Detection. *Microporous Mesoporous Mater.* **2022**, *335*, 111826, doi:10.1016/j.micromeso.2022.111826.

18. Liyanage, P.; Weerathunge, P.; Singh, M.; Bansal, V.; Ramanathan, R. L-Cysteine as an Irreversible Inhibitor of the Peroxidase-Mimic Catalytic Activity of 2-Dimensional Ni-Based Nanozymes. *Nanomaterials* **2021**, *11*, 1285, doi:10.3390/nano11051285.

19. Zhang, X.; Lu, Y.; Chen, Q.; Huang, Y. A Tunable Bifunctional Hollow Co_3O_4/MO_3 (M = Mo, W) Mixed-Metal Oxide Nanozyme for Sensing H_2O_2 and Screening Acetylcholinesterase Activity and Its Inhibitor. *J. Mater. Chem. B* **2020**, *8*, 6459–6468, doi:10.1039/D0TB01337D.

20. Zhou, X.; Wang, S.; Zhang, C.; Lin, Y.; Lv, J.; Hu, S.; Zhang, S.; Li, M. Colorimetric Determination of Amyloid-β Peptide Using MOF-Derived Nanozyme Based on Porous ZnO-Co_3O_4 Nanocages. *Microchim. Acta* **2021**, *188*, 56, doi:10.1007/s00604-021-04705-4.

21. Akhtar, R.; Yaqub, A.; Haq Khan, Z.U.; Jafry, A.T.; Ajab, H. An Octahedral Metal Oxide Nanoparticle-Based Dual-Signal Sensing Platform for Simultaneous Detection of Histidine and Lysine in Human Blood Plasma and Urine. *Nanoscale Adv.* **2025**, *7*, 1872–1884, doi:10.1039/D4NA00932K.

22. Wang, Y.; Li, W.; Liu, J.; Shi, L.; Liang, R.; Li, Y.; Sha, J. Wells-Dawson POMs Boosting Fe_2O_3 Peroxidase-Like Activity for Salicylic Acid Colorimetric Detection. *J. Inorg. Organomet. Polym. Mater.* **2024**, *34*, 6227–6235, doi:10.1007/s10904-024-03192-3.

23. Othman, H.O.; Omar, N.A.; Jabbar, H.S. CaO Nanozyme from Environmentally Friendly Waste as a Colorimetric Probe for Selective Determination of 2,4-Dichlorophenoxyacetic Acid Herbicide in Water and Soil Samples. *J. Inorg. Organomet. Polym. Mater.* **2024**, *34*, 1325–1336, doi:10.1007/s10904-023-02906-3.

24. Ma, Y.; Yi, S.; Gao, C.; Yang, M.; Feng, D.; Ren, Y.; Ge, H. Al_2O_3 -Stabilized Pt Nanozymes: Peroxidase Mimetics and Application in Glucose Detection. *ChemPlusChem* **2024**, *89*, e202300609, doi:10.1002/cplu.202300609.

25. Bharti, K.; Dalal, S.; Tyagi, A.; K. Sadhu, K. Dual Activities of Flower-Like Gold-Iron Oxide Nanozyme for Peroxidase-Mimicking and Glucose Detection. *Chem. – Asian J.* **2025**, *20*, e202401479, doi:10.1002/asia.202401479.

26. Zhang, J.; Wang, Z.; Lin, X.; Gao, X.; Wang, Q.; Huang, R.; Ruan, Y.; Xu, H.; Tian, L.; Ling, C.; et al. Mn-Ce Symbiosis: Nanozymes with Multiple Active Sites Facilitate Scavenging of Reactive Oxygen Species (ROS) Based on Electron Transfer and Confinement Anchoring. *Angew. Chem. Int. Ed.* **2025**, *64*, e202416686, doi:10.1002/anie.202416686.

27. Yao, Y.; Xu, R.; Shao, W.; Tan, J.; Wang, S.; Chen, S.; Zhuang, A.; Liu, X.; Jia, R. A Novel Nanozyme to Enhance Radiotherapy Effects by Lactic Acid Scavenging, ROS Generation, and Hypoxia Mitigation. *Adv. Sci.* **2024**, *11*, 2403107, doi:10.1002/advs.202403107.

28. Du, J.; Wang, Z.; Wang, Q.; Gu, X.; Gao, X.; Wei, H. T_2 Occupancy as an Effective and Predictive Descriptor for the Design of High-Performance Spinel Oxide Peroxidase-like Nanozymes. *Angew. Chem. Int. Ed.* **2025**, *64*, e202421790, doi:10.1002/anie.202421790.

29. Gai, S.; Chen-Wang; Jiao, W.; Teng, B.; Chi, Y.; Wang, Y.; Liu, Y.; Sun, L.; Xing, Y.; Bai, F. From Mn-Triazine Crystalline Framework to MnO_x@NC: Biomimetic Double-Enzyme Activity and Labeling of Ascorbic Acid as Well as Application in Pharmaceuticals. *Chin. J. Chem.* **2025**, *43*, 508–516, doi:10.1002/cjoc.202400938.

30. Xing, Y.; He, P.; Wang, D.; Liang, Y.; Gao, X.; Hou, X. Bimetallic Fe_3O_4@Co_3O_4/CN as a Nanozyme with Dual Enzyme-Mimic Activities for the Colorimetric Determination of Mercury(II). *Chemosensors* **2024**, *12*, 104, doi:10.3390/chemosensors12060104.

31. Qiu, Y.; Cheng, T.; Yuan, B.; Yip, T.Y.; Zhao, C.; Lee, J.; Chou, S.; Chen, J.L.; Zhao, Y.; Peng, Y. One-Pot and Gram-Scale Synthesis of Fe-Based Nanozymes with Tunable O_2 Activation Pathway and Specificity Between Associated Enzymatic Reactions. *Small* **2025**, *21*, 2408609, doi:10.1002/smll.202408609.

32. Artiglia, L.; Agnoli, S.; Paganini, M.C.; Cattelan, M.; Granozzi, G. TiO_2 @CeO_x Core-Shell Nanoparticles as Artificial Enzymes with Peroxidase-Like Activity. *ACS Appl. Mater. Interfaces* **2014**, *6*, 20130–20136, doi:10.1021/am5057129.

33. Wang, K.; Song, J.; Duan, X.; Mu, J.; Wang, Y. Perovskite $LaCoO_3$ Nanoparticles as Enzyme Mimetics: Their Catalytic Properties, Mechanism and Application in Dopamine Biosensing. *New J. Chem.* **2017**, *41*, 8554–8560, doi:10.1039/C7NJ01177F.

34. Wang, X.; Cao, W.; Qin, L.; Lin, T.; Chen, W.; Lin, S.; Yao, J.; Zhao, X.; Zhou, M.; Hang, C.; et al. Boosting the Peroxidase-Like Activity of Nanostructured Nickel by Inducing Its 3+ Oxidation State in $LaNiO_3$ Perovskite and Its Application for Biomedical Assays. *Theranostics* **2017**, *7*, 2277–2286, doi:10.7150/thno.19257.

35. Song, N.; Yu, Y.; Zhang, Y.; Wang, Z.; Guo, Z.; Zhang, J.; Zhang, C.; Liang, M. Bioinspired Hierarchical Self-Assembled Nanozyme for Efficient Antibacterial Treatment. *Adv. Mater.* **2024**, *36*, 2210455, doi:10.1002/adma.202210455.

36. Dong, H.; Du, W.; Dong, J.; Che, R.; Kong, F.; Cheng, W.; Ma, M.; Gu, N.; Zhang, Y. Depletable Peroxidase-like Activity of Fe3O4 Nanozymes Accompanied with Separate Migration of Electrons and Iron Ions. *Nat. Commun.* **2022**, *13*, 5365, doi:10.1038/s41467-022-33098-y.

37. Tian, Z.; Yao, T.; Qu, C.; Zhang, S.; Li, X.; Qu, Y. Photolyase-Like Catalytic Behavior of CeO_2. *Nano Lett.* **2019**, *19*, 8270–8277, doi:10.1021/acs.nanolett.9b03836.

38. Ragg, R.; Schilmann, A.M.; Korschelt, K.; Wieseotte, C.; Kluenker, M.; Viel, M.; Völker, L.; Preiß, S.; Herzberger, J.; Frey, H.; et al. Intrinsic Superoxide Dismutase Activity of MnO Nanoparticles Enhances the Magnetic Resonance Imaging Contrast. *J. Mater. Chem. B* **2016**, *4*, 7423–7428, doi:10.1039/C6TB02078J.

39. Pan, Y.; Zhang, Z.; Cun, J.-E.; Fan, X.; Pan, Q.; Gao, W.; Luo, K.; He, B.; Pu, Y. Oxidase-like Manganese Oxide Nanoparticles: A Mechanism of Organic Acids/Aldehydes as Electron Acceptors and Potential Application in Cancer Therapy. *Nanoscale* **2024**, *16*, 2860–2867, doi:10.1039/D3NR05127G.

40. Luo, Y.; Luo, H.; Zou, S.; Jiang, J.; Duan, D.; Chen, L.; Gao, L. An In Situ Study on Nanozyme Performance to Optimize Nanozyme-Strip for Aβ Detection. *Sensors* **2023**, *23*, 3414, doi:10.3390/s23073414.

41. Zhang, Z.; Zhang, T.; Dai, H.; Hui, Z.; Wang, L.; An, J.; Liu, X.; Shan, H.; Zheng, H.; Sun, G. Manganese Oxide (MnO_x) as Peroxidase-Mimicking Nanozymes with Valence-Dependent Activity for Single-Use Colorimetric Bioassays. *Sci. Rep.* **2025**, *15*, 42163, doi:10.1038/s41598-025-26164-0.

42. Ghaffari, M.; Nilsson, M.; Tolker-Nielsen, T.; Taheri-Kafrani, A. Peroxidase-Mimicking Chitosan/Dextran-Coated Cobalt Ferrite-Graphene Oxide Nanozyme Enhances

Hydrogen Peroxide–Mediated Killing of Cariogenic Pathogens in Dental Biofilm Models. *Sci. Rep.* **2026**, *16*, 17484, doi:10.1038/s41598-026-43315-z.

43. Péter, T.; Takács, D.; Viczián, D.; Katana, B.; Alsharif, N.B.; Szilagyi, I. Correlations Between Colloidal Stability and Peroxidase Activity of Prussian Blue Nanozymes in Salt Solutions. *J. Phys. Chem. B* **2025**, *129*, 6879–6885, doi:10.1021/acs.jpcb.5c01256.

44. Panferov, V.G.; Zhang, W.; D'Abruzzo, N.; Wang, S.; Liu, J. Kinetic Profiling of Oxidoreductase-Mimicking Nanozymes: Impact of Multiple Activities, Chemical Transformations, and Colloidal Stability. *ACS Nano* **2024**, *18*, 34870–34883, doi:10.1021/acsnano.4c12539.
